# Emergency physicians’ and nurses’ perception on the adequacy of emergency calls for nursing home residents: a non-interventional prospective study

**DOI:** 10.3389/fmed.2024.1396858

**Published:** 2024-06-19

**Authors:** Sabine Lemoyne, Joanne Van Bastelaere, Sofie Nackaerts, Philip Verdonck, Koenraad Monsieurs, Sebastian Schnaubelt

**Affiliations:** ^1^Department of Emergency Medicine, Antwerp University Hospital, Edegem, Belgium; ^2^Antwerp Surgical Training, Anatomy and Research Centre (ASTARC), University of Antwerp, Antwerp, Belgium; ^3^Department of Public Health and Primary Care, Catholic University of Leuven, Leuven, Belgium; ^4^Department of Emergency Medicine, Medical University of Vienna, Vienna, Austria; ^5^Emergency Medical Service Vienna, Vienna, Austria

**Keywords:** emergency medicine, prehospital emergency medicine, emergency medical services, emergency physician, nursing home, advance directive

## Abstract

**Introduction:**

A considerable percentage of daily emergency calls are for nursing home residents. With the ageing of the overall European population, an increase in emergency calls and interventions in nursing homes (NH) is to be expected. A proportion of these interventions and hospital transfers may be preventable and could be considered as inappropriate by prehospital emergency medical personnel. The study aimed to understand Belgian emergency physicians’ and emergency nurses’ perspectives on emergency calls and interventions in NHs and investigate factors contributing to their perception of inappropriateness.

**Methods:**

An exploratory non-interventional prospective study was conducted in Belgium among emergency physicians and emergency nurses, currently working in prehospital emergency medicine. Electronic questionnaires were sent out in September, October and November 2023. Descriptive statistics were used to analyze the overall results, as well as to compare the answers between emergency physicians and emergency nurses about certain topics.

**Results:**

A total of 114 emergency physicians and 78 nurses responded to the survey. The mean age was 38 years with a mean working experience of 10 years in prehospital healthcare. Nursing home staff were perceived as understaffed and lacking in competence, with an impact on patient care especially during nights and weekends. General practitioners were perceived as insufficiently involved in the patient’s care, as well as often unavailable in times of need, leading to activation of Emergency Medical Services (EMS) and transfers of nursing home residents to the Emergency Department (ED). Advance directives were almost never available at EMS interventions and transfers were often not in accordance with the patient’s wishes. Palliative care and pain treatment were perceived as insufficient. Emergency physicians and nurses felt mostly disappointed and frustrated. Additionally, differences in perception were noted between emergency physicians and nurses regarding certain topics. Emergency nurses were more convinced that the nursing home physician should be available 24/7 and that transfers could be avoided if nursing home staff had more authority regarding medical interventions. Emergency nurses were also more under the impression that pain management was inadequate, and emergency physicians were more afraid of the medical implications of doing too little during interventions than emergency nurses. Suggestions to reduce the number of EMS interventions were more general practitioner involvement (82%), better nursing home staff education/competences (77%), more nursing home staff (67%), mobile palliative care support teams (65%) and mobile geriatric nursing intervention teams (52%).

**Discussion and conclusion:**

EMS interventions in nursing homes were almost never seen as necessary or indicated by emergency physicians and nurses, with the appropriate EMS level almost never being activated. The following key issues were found: shortages in numbers and competence of nursing home staff, insufficient primary care due to the unavailability of the general practitioner as well as a lack of involvement in patient care, and an absence of readily available advance directives. General practitioners should be more involved in the decision to call the Emergency Medical Services (EMS) and to transfer nursing home residents to the Emergency Department. Healthcare workers should strive for vigilance regarding the patients’ wishes. The emotional burden of deciding on an avoidable hospital admission of nursing home residents, perhaps out of fear for medico-legal consequences if doing too little, leaves the emergency physicians and nurses frustrated and disappointed. Improvements in nursing home staffing, more acute and chronic general practitioner consultations, and mobile geriatric and palliative care support teams are potential solutions. Further research should focus on the structural improvement of the above-mentioned shortcomings.

## Introduction

In Belgium, 5 % of elderly people (defined as 65 years and older) live in nursing homes (NH), a highly geriatric population group with a mean age of 87 years. With the ageing of the overall European population, the percentage of elderly people in society keeps on growing each year, with a predicted increase for care-dependent elderly residing in NHs in the foreseeable future (1). So, Emergency Medical Services (EMS) response systems and Emergency Departments (ED) are already confronted with an increase in EMS calls and interventions (2–4). At this moment, almost 50% of daily emergency calls are for people aged 65 years or older, with a considerable percentage of these calls being for nursing home residents (NHRs). This poses a challenge for emergency care as NHRs are characterized by multimorbidity and frailty and hospital admissions are more frequent among NHRs when compared to community dwelling elderly, with more than 60% of transfers to ED resulting in a hospital admission, as confirmed by Carron et al. (2, 3, 5–14). In addition, transferring NHRs may have adverse complications such as pressure ulcers, delirium, hospital acquired infections and iatrogenic complications (15–17). The mortality amongst these patients is high, with many invasive procedures, and extensive use of healthcare resources while it is not yet clear if the benefits outweigh the possible adverse effects (15, 18). In conclusion, it should be noted that a high proportion of these NHRs are hospitalized shortly before death (7, 19–22).

This increase in the use of already scarce resources, raises several questions: are those transfers, transportation means, and hospital admissions appropriate? Is a low-threshold activation of the highest (physician based) tier of EMS appropriate for emergency calls in NHs, and are hospital admissions the best available medical care? (8, 23) or should health care systems optimize their use and deployment of resources for NHRs? When reviewing the literature, a proportion of these interventions and transfers may be preventable and considered inappropriate by prehospital emergency medical personnel (with inappropriate emergency department transfers accounting for 4–55% of all EMS calls), soliciting the need for further investigation of the appropriateness of hospital transfers in this patient group (5, 24–26).

Over the past few years, various studies have tried to formulate a definition of appropriateness of hospital transfers, thus resulting in various different definitions ranging from ‘preventable ED transfers’, to ‘the lowest level of safe care for a patient with a specific presentation’, to lists of symptoms and conditions (5, 24–26). To aid in defining appropriateness of hospital transfers, studies and reviews have tried to uncover the factors contributing to those transfer decisions, with several factors seeming to recur, suggesting possible ways to improve appropriateness of care (5, 8, 24–26). A first factor is the patient’s autonomy, wishes and concerns: taking those into account could raise the appropriateness of hospital transfers, while the patient’s wishes are clarified using advance directives and end of life planning. A second factor is the availability of general practitioners (GPs): with more available GPs, more NHRs could theoretically be evaluated by a physician before hospital transfer, and this could concomitantly enhance the appropriateness of transfers and reduce the numbers of EMS calls. A third factor is the education and availability of NH staff: with rising levels of education and staff availability, the appropriateness of hospital transfers could improve. A fourth factor is the possibility to provide acute care in the NH whenever possible, reducing the need for hospital transfers. Lastly, better interprofessional collaboration and communication could improve the appropriateness of hospital transfers.

Studies have also tried to shed light on the perspectives of NH staff, GPs and family members on hospital transfers of NHRs to define the appropriateness of hospital transfers (27–29). Oppositely, the perspectives of the prehospital emergency medical personnel have never been studied, leaving a gap in today’s literature. Therefore, the primary aim of this study was to unveil the perspectives of Belgian prehospital emergency medical personnel on the appropriateness of emergency calls and interventions in NHs. How often are EMS calls perceived as indicated or necessary? What do health care professionals regard as an adequate activation and use of EMS resources for NHRs? The secondary aim of this study was to explore the factors contributing to their perspectives, and to formulate possible solutions to improve the adequacy of EMS calls for NHRs.

## Materials and methods

### Study design and population

An exploratory non-interventional prospective study (see Supplementary Table S15) was conducted in Belgium among 114 Emergency Physicians (EPs) and 78 Emergency Nurses (ENs) (30). An electronic questionnaire was developed in an expert consensus approach by the authors of this paper. Starting from the original English version, the authors translated the questionnaire into Dutch (Flemish) and French to fit the respective focus groups. Hereafter, the questionnaire was reviewed by a small number of test respondents (physicians), before finalization. Only EPs and ENs with prehospital experience were targeted. The questionnaire was sent out to the majority (126) of Belgian emergency departments on the 9th of September 2023, as a Google form in both languages (31). A second wave was sent out on the 17th of October 2023 and a final wave on the 1st of November 2023. The emergency medicine related organizations BeSEDiM (Belgian Society of Emergency and Disaster Medicine), BeCEP (Belgian College of Emergency Physicians) and VAUG (Vlaamse Academie voor Urgentiegeneeskunde) also spread the questionnaire among their members. All questionnaires were filled out completely anonymously and written informed consent was obtained. The study obtained a positive evaluation by the Ethics Committee of the Antwerp University Hospital (UZA; Project no. 5509) on the 3rd of July 2023.

The questionnaire (see Supplementary Tables S1–S6) asked the respondents to give their opinion concerning diverse topics on the EMS interventions in NHs. Of note, the respondents were asked to not report on their last intervention or shift, but rather give an estimated mean of the remembered past interventions. Specifically, the questionnaire contained topics covering demographics of the respondents, general and specific characteristics of the interventions (including involvement of GPs), interprofessional communication, availability of and respect for advance directives, medical treatment provided by EMS personnel and emotional aspects of the EMS team. Possible differences in perceptions between EPs and ENs were evaluated regarding general and specific characteristics of EMS interventions in NHs, medical interventions, advance directives and emotions (see Supplementary Tables S7–S11). The Likert scales used for answers in frequency, adequacy and agreement are listed in Table 1.

**Table 1:** Likert scales and values for questionnaire answers in frequency, adequacy and agreement.

**Likert scales and values**		
Frequency Likert Scale	Adequacy Likert Scale	Agreement Likert Scale
1:Never 2:Almost never 3:Sometimes 4:Often 5:Almost always	1:Very inadequate 2:Inadequate 3:Neutral 4:Adequate 5:Very adequate	1:I do not agree at all 2:I do not agree 3:Neutral 4:I agree 5:I fully agree

Data of returned questionnaires was entered into a standardized electronic case report form, and statistical analyses (where eligible) were carried out via SPSS (Version 26.0, IBM). Means and standard deviations were calculated for continuous variables. Absolute and relative frequencies, as well as medians and interquartile ranges, were used to express categorical and nominal variables. Group comparisons (responses of ENs vs. EPs) were conducted using the *χ*^2^ test. Correlations were calculated by using the methods of Pearson, Fisher’s exact and Mann–Whitney-Wilcoxon. For binary independent variables, Pearson *χ*^2^ test and Fisher’s exact test were used. For analyses of the results of Likert scales, Pearson *χ*^2^ test, Fisher’s exact test and Mann–Whitney-Wilcoxon test were used. A two-sided *p*-value of <0.05 was considered significant. As this is an exploratory study, *a priori* sample size calculation was not performed.

### EMS in Belgium

Emergency Medical Services (EMS) refer to a comprehensive system designed to provide emergency medical care outside of a hospital setting. EMS encompasses various components, such as ambulance services, dispatch systems, medical oversight, and coordination with hospitals, with the primary goal of stabilizing patients and transporting them to appropriate healthcare facilities for further evaluation and treatment.

EMS intervention refers to the immediate medical actions taken by trained professionals in response to a medical emergency. These interventions aim to provide timely and appropriate medical care to individuals experiencing acute illness or injury outside of a hospital setting. EMS interventions may include assessing and stabilizing vital signs, administering medications, performing advanced medical procedures such as airway management and cardiopulmonary resuscitation (CPR), immobilizing fractures, controlling bleeding, and providing emergency transportation to a medical facility for further evaluation and treatment. The goal of EMS interventions is to minimize morbidity and mortality by rapidly addressing life-threatening conditions and optimizing patient outcomes.

Medical emergencies are defined as occurring suddenly and unexpectedly, often requiring prompt action from trained healthcare professionals, including emergency medical technicians (EMTs), paramedics, and emergency physicians. Access to emergency medical services (EMS) plays a crucial role in providing timely care and transportation to appropriate medical facilities for further evaluation and treatment.

The Belgian EMS is a governmentally regulated system started in 1959 and further developed to its current system since 2005. It consists of a four-tier based system, with eight levels of care (see Supplementary Table S14). All participating tiers are flat-rate funded by the government.

A centralized dispatch centre regulates every EMS call using a protocol-based system, established on the assessment of the patient’s level of consciousness, breathing and circulation. After the initial evaluation of the severity of the EMS call, the dispatch centre activates the relevant severity level and therefore needed tier of assistance. When an airway, breathing or circulation problem is suspected, the highest tier is automatically activated.

The first tier consists of a GP-mediated (non-emergent) intervention and covers level 6, 7 and 8. At level 6, medical assessment by a GP is needed within the next 2 h. At level 7, this is needed within the next 12 h and at level 8, medical evaluation can be postponed to assessment by the patient’s own GP outside of the on-call setting. In Belgium, GPs are self-employed, but legally and governmentally bound to participating in the on-call duty.

An ambulance staffed with two paramedics constitutes the second level and covers level 5, being a non-life-threatening situation where a swift transfer to the hospital is needed for further diagnosis and treatment. Ambulances can be private or non-private, but participation in EMS interventions is governmentally bound.

The third level, known as PIT (Paramedical Intervention Team), consists of an ambulance with an EN and a paramedic, and covers levels 3 and 4. At level 3, the situation may shortly evolve into a life- or organ-threatening situation needing urgent medical assistance. At level 4, there is no need for urgent medical assistance. A PIT is always non-private and hospital bound.

The fourth tier, known as a MUG (Mobile Urgency Group), is a team consisting of an EP and an EN and covers levels 1 and 2. Level 1 is a life-threatening situation, whereas level 2 is a potentially life-threatening situation needing emergent medical care. In analogy to a PIT, a MUG is always non-private and hospital bound. Emergency physicians in Belgium are highly educated, with 6 years of specific training in emergency medicine.

If necessary, each tier can activate a second team or a higher-leveled team. The dispatch centre does not have adapted protocols for NHRs, and this issue has never been established or even discussed before due to organizational issues and ethical difficulties (32).

## Results

### Overall respondent characteristics

A total of 192 respondents (114 or 59% EPs and 78 or 41% ENs) returned the questionnaire. The characteristics of the respondents are shown in Supplementary Table S1. The mean age was 38 (± 10) years, 51% were male, 70% worked in urban-, 46% in suburban-, and 26% in rural areas (with the possibility of working in more than one type of area). Less than half (36%) of responding EPs were emergency physician residents, with the rest thus having a concluded training in emergency medicine. Almost all (96%) ENs had followed a postgraduate education in emergency medicine and/or intensive care after their bachelor’s degree in nursing. The working experience of all respondents was 13 (±9) years in healthcare, and 10 (± 9) years in prehospital emergency care. Only 18% had training in geriatric care or geriatric emergencies. Forty-five percent of the respondents expressed an interest in additional geriatric emergency training.

### Perceptions on EMS nursing home interventions

Respondents estimated that, per 12-h shift, there were a mean of 1.3 (± 0.9) NH interventions of a total of 3.4 (± 1.8) overall interventions per 12-h shift. Additionally, 62% felt that there were more EMS interventions in NHs during weekends and nights.

Almost all (90%) respondents reported that the NHs were understaffed, and 95% perceived the competence of the NH staff as inadequate. Dynamics in NH staffing during the weekends and nights were noticed by 97%, with 89% noticing less attending NH personnel during nights and weekends while nobody reported an increase. Sixty-eight percent of respondents believe that variations in available attending staff significantly affected the quality of care in NHs. The majority (73% of the ENs and 63% of the EPs) of the respondents felt that there should be a 24/7 availability of a GP in the NH, and most of them (66% of the EPs and 52% of the ENs) thought that a hospital admission should only be occur after consulting a GP (3.7 ± 1.2). This is in contrast to reality, as the respondents felt that there was almost never a GP present at the NH before initiation of an EMS call [2.09 (SD ± 0.68)]. The GP, NH physician or on-call physician was often unavailable, leading to activation of the EMS system [3.75 (SD ± 0.78)]. Of note, the majority of respondents (68%) felt that hospital admissions could have been avoided if NH staff had more authority in initiating medical treatments. Most of the respondents agreed that proper care by the NH staff could have reduced the necessity of medical interventions performed by an EP [3.89 (SD ± 0.72)]. The EMS team was under the impression that the reason for activating the EMS system was only sometimes due to an emergency [2.50 (SD ± 0.82)] and that the reason was often a slowly deteriorating chronic condition [3.93 (SD ± 0.73)]. In many cases the real reason for an EMS call was the mere need for a transfer to the hospital without emergency [3.21 (SD ± 1.04)]. Further details on EMS NH interventions and respective medical interventions are shown in Table 2 and Supplementary Tables S2, S3.

**Table 2:** An example of the questions and topics with the respondents’ mean answers according to the Likert scales for frequency, adequacy, and agreement as described in table.

**Question/topic**	**Likert scale**
How often is there telephone contact with the GP prior to the EMS call?	2.7 ± 1.0
How often is the GP present before the initiation of an EMS call?	2.1 ± 0.7
How often is an EMS call following the patient’s wishes?	2.2 ± 0.7
How often is an EMS call at request of the patient or the patient’s family?	2.4 ± 0.9
How often are EMS interventions in the NH necessary or indicated?	2.3 ± 0.6
How often is an EMS call due to an emergency?	2.5 ± 0.8
How often is an EMS call due to a slowly deteriorating chronic condition?	3.9 ± 0.7
How often is an EMS call a mere request for hospital transfer (without emergency)?	3.2 ± 1.0
How often is the presence of an EP necessary or indicated?	1.9 ± 0.6
How often is the right EMS level activated?	2.5 ± 0.9
How often is the reason of activation clear to you on arrival?	2.6 ± 0.9
How often is there a professional handover of information on arrival?	2.3 ± 0.9
How often do you receive all important documentation from the NH personnel?	2.9 ± 1.1
How often is there adequate first aid response in the NH in case of emergency?	2.4 ± 0.9
How often are medical interventions performed by the EMS team during NH interventions?	3.1 ± 1.0
How often are medical interventions performed by an EP during NH interventions?	2.7 ± 1.0
How often could such interventions be avoided by more proper care by the NH?	3.9 ± 0.7

For the rest of the questions/topics, see Supplementary material.

GP, general practitioner; EMS, emergency medical services; NH nursing home; EP, emergency physician.

The respondents were also asked to define avoidable hospital admissions in free text answers. All answers are shown in Supplementary Table S12.

### Ethical issues and advance directives

Ninety-two percent of respondents agreed that it is legally possible to avoid hospital admission by leaving the resident at the NH. Ninety-four percent reported that the EP could theoretically make this decision. Nevertheless, the NHR is almost always transferred to an ED (4.4 ± 0.67), which is perceived as frequently not in accordance with the patients’ wishes (2.5 ± 0.7).

Regarding advance directives, 67% of respondents reported to have had specific training on this topic, and 71% were under the impression to be legally bound by an advance directive if readily available. Those advance directives were, however, perceived as almost never being present (2.1 ± 0.7). Another feeling was that EMS calls were often placed for Advanced Life Support despite the availability of a negative advanced directive (3.6 ± 0.9). Furthermore, respondents reported that the decision to transfer a NHR to the ED with an advanced medical condition should be taken more cautiously (4.5 ± 0.7), as well as the decision to transfer a NHR suffering from advanced dementia (4.5 ± 0.8).

Palliative care in NHs was perceived as inadequate in many cases (2.2 ± 1.0), and chronic conditions often as un- or undertreated (3.6 ± 0.9), with specifically chronic pain being un- or undertreated (3.2 ± 0.8). When admitting a NHR to a hospital, many EPs and ENs felt disappointed (51%) and frustrated (61%). The EMS team was more afraid of the medical implications by doing too little [2.75 (SD ± 1.21)], than they were of the medical implications by doing too much [2.39 (SD ± 1.21)].

Interestingly, it was reported that an EMS call often seems to redirect the responsibility for the NHRs’ care from the NH staff toward someone else (4.0 ± 0.9), and that hospital admission of the NHR often seems to redirect the responsibility for the NHRs’ care from the EMS team toward someone else (probably an ED; 3.8 ± 0.9).

Further details regarding ethics and advance directives are shown in Supplementary Table S4, and further details regarding respondents’ emotions in Supplementary Table S5.

### Suggested solutions

The respondents made several suggestions to reduce the number of NH EMS calls and to reduce ED transfers: more GP involvement (82%), better NH staff education/competences (77%), more NH staff (67%), mobile palliative care support teams (65%), mobile geriatric nursing intervention teams (52%), specific EMS tiers designed for NH interventions (31%), and telemedicine consultations (20%). The respondents’ free text answers regarding possible medical interventions that could avoid hospital admissions were the administration of intravenous medication (such as antibiotics, antipyretics, diuretics and fluids), oxygen therapy, palliative care, aerosols and adequate pain management. All answers to the open questions are listed in Supplementary Tables S9, S10 in their original form.

### Differences between EPs and ENs

A comparison was made between the answers of EPs and ENs. All respective data are shown in Supplementary Tables S6–S8. ENs indicated that the NH physician should be available 24/7 while EPs felt less strongly about this (*p* < 0.001) (Supplementary Figure S1). There was a significant difference between their perception regarding the possible avoidance of hospital admissions if NH staff had more authority concerning the initiation of medical interventions (73% of ENs vs. 40% of EPs thought hospital admissions could be avoided by more NH staff authorities, *p* < 0.001). EN felt more strongly that the patients’ wishes were not followed in comparison to EP, especially regarding the placement of the EMS call (*p* = 0.021) (Supplementary Figure S2), but also during an EMS intervention (*p* < 0.001) (Supplementary Figure S3). ENs were under the impression that there was more inadequate pain management in the NH than EPs (*p* = 0.006) (Supplementary Figure S4). EPs were more afraid of the medical implications by doing too little compared to ENs (*p* = 0.048) (Supplementary Figure S5), while EPs were less afraid of overtreatment (*p* = 0.015) (Supplementary Figure S6). Both ENs and EPs had the perception that the EMS call was almost never due to an acute emergency, but ENs felt more strongly about this (*p* = 0.031) (Supplementary Figure S7). Both ENs and EPs had the perception that the EMS call was often for Advanced Life Support in spite of a negative advance directive, although the EPs felt more strongly about this than the ENs (*p* = 0.014) (Supplementary Figure S8). ENs thought more often that the EMS paramedic team (PIT) was activated appropriately compared to EPs (*p* = 0.01) (Supplementary Figure S9).

## Discussion

This exploratory non-interventional prospective study gives an overview of the perception of EPs and ENs on EMS calls for NHRs in Belgium. The respondents estimated that around one third of all EMS interventions during a 12-h shift are in NH, making them quite frequent. EMS interventions consume budget and personnel resources. The feedback from the EMS staff involved, could contribute toward an optimization of the use of EMS resources for NHRs.

The primary aim of this study was to unveil the perspectives of Belgian EPs and ENs toward the appropriateness of emergency calls and interventions in NH. Our data suggests that EMS interventions in NHs are almost never perceived as necessary or indicated, with the right EMS level almost never being activated. Especially MUG teams are often unjustly activated, with the presence of an EP almost never being necessary or indicated. The activation of PIT teams is perceived as being more adequate, with only sometimes being unjustly called for in the perception of EPs and almost never being unjustly called for in perception of ENs. This shows that especially physician-based EMS tiers are being perceived as inappropriate by prehospital emergency medical personnel. EMS teams in Belgium are often overqualified for the NH interventions they are sent to. One should reflect on downscaling the tier for NH interventions (8, 23).

To further define appropriateness of EMS calls and interventions, the secondary aim of this study was to explore the factors contributing to the perception of inappropriateness of those calls and interventions. What about the nursing home residents: are their wishes, autonomy and concerns considered? What about the NH staff and the GPs who should – in theory – be the first point of medical contact? The results of our survey are in accordance with other authors and indicate several structural reasons for activating the EMS system. To cite the existing literature, inaccessible primary care and shortages in NH staff lead to high numbers of EMS activations (5, 28, 33–35). An important contributing factor to avoidable transfers is the lacking involvement of the GPs (27, 28, 33). Briggs et al. reported that in up to 40% of the ED transfers there was no prior review by any physician and this number increased up to 77% outside of ‘normal’ working hours (36). Pulst et al. reported that GPs were involved in only 34.8% of transfer decisions (5). Our study suggests that there does not only seem to be a lack of involvement from the GPs prior to the EMS call, there also seems to be a lack of involvement in the chronic and daily care for NHRs leading to more EMS calls. Prior to an EMS call, there was only sometimes telephonic contact with a GP. The GP was almost never present in the NH before the initiation of the EMS call and a GP placed the EMS call in only 11.5% of the cases. Secondly, EMS calls were often due to unavailability of the GP, NH physician or on-call physician. Regarding this statement, 73% of ENs were convinced that NH physicians should be available 24/7, whilst 52% of EPs had the same appreciation. Thirdly, EMS interventions were perceived as almost never acute emergencies considering that the reason for EMS calls was often a slowly deteriorating chronic condition and NHRs often had un- or undertreated chronic conditions and sometimes un- or undertreated pain. This shows that there is room for improvement of the chronic and daily care for NHRs by the GPs, and room for improvement in the involvement of the GPs in not only the chronic but also acute care for NHRs. More GP involvement is indispensable when trying to avoid inappropriate transfers. This is an important structural measure to be addressed.

Moreover, there seem to be challenges in NH staffing: according to over 90% of respondents, NH are not adequately staffed in numbers and over 95% believe that NH are not adequately staffed in competence. Furthermore, there seems to be an increase of EMS interventions during weekends and nights, possibly because there is less personnel (88.8% of respondents) and less competent personnel (64.2% of respondents) at that time, affecting the quality of care. This is in accordance with von der Warth et al., who concluded that understaffing is one of the biggest problems in NHs (37). During those EMS interventions, there are sometimes avoidable acute medical interventions performed by members of the EMS team, with 68.2% of respondents thinking that hospital admissions could be avoided if NH staff had more authority concerning the initiation of such acute medical interventions. Also, there seem to be an issue with competences regarding first aid and palliative care provided by the NHs. This is in accordance with Miller et al., who concluded that the introduction of palliative care consultations in NHs is associated with overall reduction of end-of-life hospital admissions (38). Like Smets et Al. we also found that palliative care and pain treatment are perceived as insufficient in NHs (39). In conclusion, this suggests that there is room for improvement of NH staff in numbers, in competence (and thus education) and in authority. This could improve the chronic and acute care in NHs and reduce the number of EMS interventions, with a diminished need for hospital transfers. Mobile geriatric nursing intervention teams could contribute to the solution. They can support NH staff with specific geriatric clinical expertise and skills. Of course, there needs to be sufficient staff and more specific NH staff education. Concomitantly, NHRs in their last phase of life could benefit of mobile palliative care support teams as these interventions likely meet the needs of the NHRs so that they can remain at their current place of residence (40). Piot et al. (40) concluded that the activity of mobile palliative care support teams remains marginal, although steadily on the rise. These teams aim to relieve suffering and improve quality of life by addressing the physical, psychosocial, and spiritual needs of NHRs in their end-of-life period (41).

Regarding ethics, communication and interprofessional collaboration, EPs and ENs state that the reason for the EMS interventions was only sometimes clear on arrival, and that they almost never receive a clear handover of information by NH staff. These findings were already stated by other authors (27, 28, 37, 42–45). Respondents also reported that NHs almost never have advance directives of their residents, and concomitantly often call for Advanced Life Support despite negative advance directives. Other authors confirmed the lack of advance directives (45–47). This is a missed opportunity to avoid inappropriate hospital transfers and care. Almost all respondents feel that we need to reflect more cautiously when considering transfer of a NHR with an advanced medical condition or advanced dementia. Hospital admissions should preferably be initiated after contact with the GP, thus underlining the importance of interprofessional collaboration and communication. Many respondents felt that the activation of the EMS system and the request to transfer the NHR to the hospital were not in accordance with the resident’s wishes. This is also reported by Morphet et al. (48) and is very disturbing. Notably, hospital admissions do not necessarily result in better care. Many patients experience distress and/or discomfort, and even delirium occurs when they are taken out of their familiar environment. Vulnerable elderly persons in their last phase of life are transported to a hectic ED, and ED visits put these patients at risk of increased morbidity, hospital readmission and death (48, 49).

Of note, the often hectic course of an EMS intervention does not always provide enough time for EMS teams to fully assess a NHRs’ situation, and it can be difficult to perceive a patient’s real wishes when chronically or acutely severely ill. Moreover, our respondents reported that NH staff tend to redirect the responsibility for the NHRs’ care from the NH staff toward someone else, potentially building pressure toward a quick hospital admission (50). Also, the decision to hospitalize a NHR might be mediated by a fear for medico-legal consequences if one does too little (as stated by our study respondents), thus redirecting the responsibility for the NHRs’ care from themselves onto the next health care provider. Additionally, in spite of our respondents reporting feelings of “frustration” and “disappointment” when hospitalizing an NHR, they often do so, possibly in an attempt to compensate for the failing care in the NH. Henckes and Nurok (51) stated that the management of emotions is particularly important for group dynamics in EMS and their functioning. They developed a framework for analyzing the collective management of emotions at work in EMS (51). Until now reforming the EMS deployment to NHs has not been discussed in Belgium. The ethical considerations surrounding EMS interventions in NHs are multifaceted and require careful examinations from various perspectives. Several aspects need to be addressed: autonomy of the NH resident, beneficence and non-maleficence by the healthcare professionals, quality of life of the NH resident, communication and collaboration, advance care planning and cultural and religious considerations. Other important aspects are cost-effectiveness, resource allocation and managing healthcare expenditure.

Besides trying to define the appropriateness of EMS calls and interventions in NH, this study also tried to formulate possible solutions to improve the adequacy of EMS calls for NHRs. EPs and ENs were asked how they believe EMS calls, interventions and hospital admissions could be reduced.

Thus, in summary, optimal continuous and chronic care, paired with a functional advance directive system, seem of outmost importance to aid the EMS in taking part in shared decision making and patient-centred care. One must, however, not forget that some NHRs still have a high quality of life and fall acutely ill. Naturally, an EMS intervention and swift hospital admission is appropriate in these patients.

## Limitations

The results of this study should be interpreted with caution as the reported data are all personal and subjective impressions of the participating EPs and ENs. Furthermore, the questionnaire was developed in an expert consensus approach by the authors of this paper, therefore allowing for potential subjectivity in several of the questions asked and possible bias in construct, content and criterion validity.

Defining inappropriate ED transfers is challenging. The reasons for EMS calls and transfers of NHRs to the hospital are multiple and complex, and the designed questionnaire may not have addressed all relevant topics and issues, leaving gaps which were thus not covered (25, 52, 53). There might also be selection bias or sample bias, as respondents might have been triggered to participate in the questionnaire out of interest in the subject.

The presented *p*-values were calculated in exploratory analyses and they were not adjusted for multiple testing since sample size calculation was not originally planned for the comparison of EPs and ENs.

The perspectives of paramedics, NHRs and family were not obtained in the present study, but should be part of future research. Whereas some comparability can be suggested at least to countries in the European Union, due to organizational differences between EMS systems, our findings may not be automatically transmittable to other healthcare systems outside Belgium.

## Conclusion

In our evaluation of the perception of prehospital emergency medical personnel on EMS interventions in NHs, these interventions were almost never seen as necessary or indicated by EPs and ENs, with the right EMS tier almost never being activated. Especially the (over-)use of physician-based tiers is being perceived as inappropriate. Inaccessible primary care, shortages in NH staff and lacking involvement of the GPs in both acute and chronic care for NHRs, led to high numbers of inappropriate EMS calls and interventions. Hospital admission should preferably be initiated after contact with a GP, thus underlining the importance of interprofessional collaboration and communication. Advance directives are almost never available, not only leading to decisions not following the patient’s wishes, but also leading to requests for Advanced Life Support despite potentially negative directives. Healthcare workers should therefore strive for vigilance regarding the patient’s wishes. The emotional burden of deciding on an avoidable hospital admission of NHRs, perhaps out of fear for medico-legal consequences if doing too little, leaves the EPs and ENs mostly frustrated and disappointed.

Respondents perceived that an increase in NH staff numbers and better education, more acute and chronic GP consultations, mobile geriatric nursing intervention teams and mobile palliative care support teams could contribute to reducing the amount of avoidable EMS interventions in NHs.

Further research should strive to validate our findings in paramedics, NHRs, and family members, and focus on long-term interventions to structurally improve the above mentioned shortcomings.

## Data availability statement

The original contributions presented in the study are included in the article/Supplementary material, further inquiries can be directed to the corresponding author.

## Author contributions

SL: Conceptualization, Investigation, Methodology, Supervision, Validation, Writing – original draft, Writing – review & editing. JB: Conceptualization, Data curation, Formal analysis, Methodology, Writing – original draft, Writing – review & editing. SN: Data curation, Formal analysis, Writing – original draft, Writing – review & editing. PV: Writing – original draft, Writing – review & editing. KM: Writing – original draft, Writing – review & editing. SS: Conceptualization, Methodology, Supervision, Validation, Writing – original draft, Writing – review & editing.
