# Supplementary material for: Emergency physicians’ and nurses’ perception on the adequacy of emergency calls for nursing home residents: a non-interventional prospective study
Source: Front Med (Lausanne). 2024 Jun 19;11:1396858. doi: 10.3389/fmed.2024.1396858 (PMC11220277; doi:10.3389/fmed.2024.1396858)
Supplement: Supplementary file 1 [file Data_Sheet_1.PDF]

Emergency physicians‘ & nurses‘ perception on the adequacy of emergency calls for nursing home residents

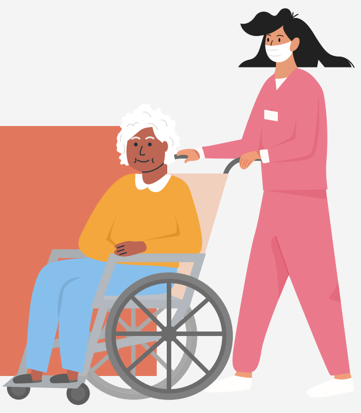

- *A CONSIDERABLE AMOUNT OF EMERGENCY CALLS IS FOR NURSING HOME RESIDENTS*
- *A NUMBER OF THEM MIGHT BE PREVENTABLE*

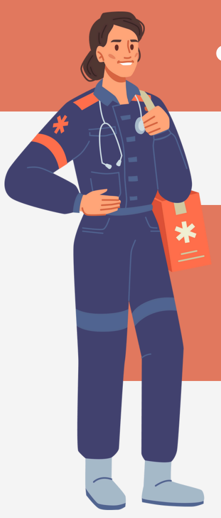

OPINIONS & PERCEPTIONS OF EMS PERSONNEL ABOUT EMS MISSIONS AND INTERVENTIONS IN NURSING HOMES ARE VALUABLE

- Non-interventional prospective study in Belgium
- Questionnaires for EMS physicians and EMS nurses

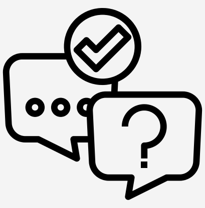

- 114 EMS-PHYSICIANS AND 78 EMS-NURSES RESPONDED; “DISAPPOINTED“ & “FRUSTRATED“

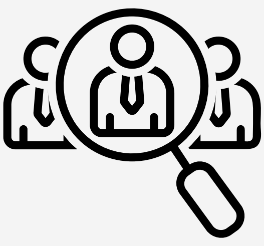

- NURSING HOME STAFF PERCEIVED AS UNDERSTAFFED & LACKING IN COMPETENCE
- GENERAL PRACTITIONERS PERCEIVED AS INSUFFICIENTLY INVOLVED & OFTEN UNAVAILABLE

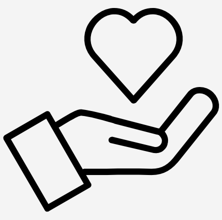

- ADVANCE DIRECTIVES ALMOST NEVER AVAILABLE
- HOSPITALIZATIONS POTENTIALLY NOT ACCORDING TO PATIENTS’ WISHES
- PALLIATIVE CARE & PAIN TREATMENT INSUFFICIENT

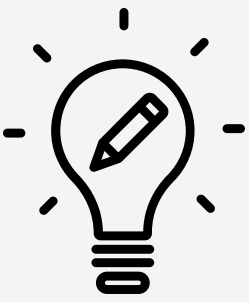

- SUGGESTIONS TO REDUCE EMS INTERVENTIONS:
  - MORE GENERAL PRACTITIONER INVOLVEMENT (82%)
  - BETTER NURSING HOME STAFF EDUCATION AND COMPETENCES (77%)
  - MORE NURSING HOME STAFF (67%)
  - MOBILE PALLIATIVE CARE SUPPORT TEAMS (65%)
  - MOBILE GERIATRIC TEAMS (52%)

CONCLUSIONS

- EMS interventions in nursing homes almost never seen as indicated & appropriate EMS tier almost never activated.
- Shortages in numbers and competence of nursing home staff, insufficient primary care, absence of readily available advance directives & mobile geriatric and palliative care.
- General practitioners should be more involved in the decision to call EMS.
- Healthcare workers should strive for vigilance regarding patients’ wishes.
- Further research should focus on structural improvements.

CITATION

Lemoyne S, Van Bastelaere J, Nackaerts S, Verdonck P, Monsieurs KG, Schnaubelt S. Emergency physicians’ and nurses’ perception on the adequacy of emergency calls for nursing home residents: a non-interventional prospective study. Frontiers in Medicine 2024
