# Supplementary material for: Emergency physicians’ and nurses’ perception on the adequacy of emergency calls for nursing home residents: a non-interventional prospective study
Source: Front Med (Lausanne). 2024 Jun 19;11:1396858. doi: 10.3389/fmed.2024.1396858 (PMC11220277; doi:10.3389/fmed.2024.1396858)
Supplement: Supplementary file 2 [file Data_Sheet_2.docx]

***SUPPLEMENTARY MATERIAL***

1. **SUPPLEMENTARY TABLES**

**Supplementary Table S1:** Characteristics of the respondents

| Characteristics of the respondents | |
| --- | --- |
| Age (years), mean (± SD) | 37.64 (± 10) |
| Gender, n (%)  Man  Woman  Non-binary  Other  Missing data | 98 (51%)  93 (48.4%)  0 (0%)  0 (0%)  1 (0.6%) |
| Area of work, n (%)                Urban                Suburban                Rural | 135 (70.3%)  89 (46.4%)  50 (26%) |
| Occupation, n (%)                Physician                Nurse | 114 (59.4%)  78 (40.6%) |
| Type of training (physician), n (%)  In training  Specialist - Emergency Medicine  Does not apply | 41 (36%)  71 (62.1%)  2 (1.8%) |
| Type of training (nurse), n (%)  Bachelor nursing  Postgraduate Emergency Medicine and/or Intensive Care  Bachelor After Bachelor Care Management  Other  Does not apply | 1 (1.3%)  75 (96.1%)  1 (1.3%)  1 (1.3%)  0 (0%) |
| Years of experience in healthcare (years), mean (± SD) | 13 (± 9.13) |
| Years of experience in prehospital care (years), mean (± SD) | 10 (± 8.79) |
| Training in geriatric care or geriatric emergencies, n (%)  Yes  No | 35 (18.2%)  157 (81.8%) |

**Supplementary Table S2:** Characteristics of Emergency Medical Services (EMS) interventions in nursing homes (general information)

| Characteristics of EMS interventions in nursing homes (general information) | |
| --- | --- |
| Number of EMS interventions during a 12-hour shift, mean (± SD) | 3.43 (± 1.80) |
| Number of EMS interventions in nursing homes during a 12-hour shift, mean (± SD) | 1.25 (± 0.89) |
| Are there more EMS interventions during weekends and nights, n (%)  Yes  No | 118 (61.5%)  72 (37.5%) |
| Is there adequately staffed personnel in nursing homes in numbers, n (%)  Yes  No | 16 (8.3%)  173 (90.1%) |
| Is there adequately staffed personnel in nursing homes in competence, n (%)  Yes  No | 7 (3.6%)  183 (95.3%) |
| Is there a difference in staffing between nights and weekends, n (%)  Yes  No | 186 (96.9%)  5 (2.6%) |
| Type of differences in staffing between nights and weekend, n (%)  Less personnel  More personnel  Less competent personnel  More competent personnel  Other | 166 (88.8%)  0 (0%)  120 (64.2%)  0 (0%)  6 (3.2%) |
| Does the difference in staffing impact the quality of care, n (%)  Yes  Sometimes  No | 130 (67.7%)  55 (28.6%)  2 (1%) |

EMS: Emergency Medical Services

**Supplementary Table S3**: Characteristics of EMS interventions in nursing homes (specific information):

| Characteristics of EMS interventions in nursing homes (specific information) | |
| --- | --- |
| 112-call initiator, n (%)  Personnel of nursing home  General practitioner  Patient  Family member  Other | 166 (88%)  97 (50.5%)  5 (2.6%)  30 (15.6%)  2 (1%) |
| 112-caller, n (%)  Personnel of nursing home  General practitioner  Patient  Family member  Other | 186 (96.9%)  22 (11.5%)  0 (0%)  3 (1.6%)  0 (0%) |
| How often is there telephone contact with a general practitioner before initiation of a 112-call - Frequency Likert Scale  Mean (± SD)  Median [IQR] | 2.68 (± 0.95)  3 [1] |
| How often is the general practitioner present before initiation of a 112-call - Frequency Likert Scale  Mean (± SD)  Median [IQR] | 2.09 (± 0.68)  2 [0] |
| How often is the placement of a 112-call in accordance with the patient’s wishes - Frequency Likert Scale  Mean (± SD)  Median [IQR] | 2.24 (± 0.68)  2 [1] |
| How often is a family member present on arrival of the EMS team - Frequency Likert Scale  Mean (± SD)  Median [IQR] | 1.90 (± 0.69)  2 [1] |
| How often is a 112-call due to an acute emergency - Frequency Likert Scale  Mean (± SD)  Median [IQR] | 2.50 (± 0.82)  2 [1] |
| How often is a 112-call due to a slowly deteriorating chronic condition - Frequency Likert Scale  Mean (± SD)  Median [IQR] | 3.93 (± 0.73)  4 [0] |
| How often is a 112-call a mere request for hospital transfer (without emergency) - Frequency Likert Scale  Mean (± SD)  Median [IQR] | 3.21 (± 1.04)  3 [2] |
| How often is a 112-call a request from the patient or the patient’s family (without emergency) - Frequency Likert Scale  Mean (± SD)  Median [IQR] | 2.36 (± 0.88)  2 [1] |
| How often are EMS interventions in nursing homes really necessary or indicated - Frequency Likert Scale  Mean (± SD)  Median [IQR] | 2.25 (± 0.63)  2 [1] |
| How often is the presence of an emergency physician really necessary or indicated - Frequency Likert Scale  Mean (± SD)  Median [IQR] | 1.94 (± 0.55)  2 [0] |
| How often is the appropriate EMS level activated - Frequency Likert Scale  Mean (± SD)  Median [IQR] | 2.45 (± 0.92)  2 [1] |
| How often is a EMS team with nurse (PIT) unjustly activated - Frequency Likert Scale  Mean (± SD)  Median [IQR] | 3.19 (± 0.82)  3 [1] |
| How often is a EMS team with physician (MUG) unjustly activated - Frequency Likert Scale  Mean (± SD)  Median [IQR] | 3.91 (± 0.86)  4 [0] |
| How often is the reason of activation clear to you on arrival - Frequency Likert Scale  Mean (± SD)  Median [IQR] | 2.64 (± 0.92)  3 [1] |
| How often is there a clear and professional handover of information - Frequency Likert Scale  Mean (± SD)  Median [IQR] | 2.31 (± 0.93)  2 [1] |
| How often do you receive all important documentation from the nursing home personnel - Frequency Likert Scale  Mean (± SD)  Median [IQR] | 2.94 (± 1.1)  3 [2] |
| How often is there adequate first aid in the nursing home in case of emergency - Frequency Likert Scale  Mean (± SD)  Median [IQR] | 2.38 (± 0.85)  2 [1] |
| How often are there language barriers with nursing home staff, leading to miscommunication - Frequency Likert Scale  Mean (± SD)  Median [IQR] | 2.51 (± 1.01)  2 [1] |
| Is it legally possible in your system to leave a nursing home resident at the nursing home (= not performing a hospitalization) when on an EMS mission, n (%)  Yes  No | 176 (91.7%)  16 (8.3%) |
| Who can make the decision to leave a nursing home resident at the nursing home (= not performing a hospitalization) when on an EMS mission, n (%)  Paramedic  Nurse  Physician  Other  This is not possible | 9 (4.7%)  29 (15.1%)  180 (93.8%)  30 (15.6%)  7 (3.7%) |
| How often is there transport of a nursing home resident to the hospital - Frequency Likert Scale  Mean (± SD)  Median [IQR] | 4.36 (± 0.67)  4 [1] |
| How often are hospitalizations avoidable - Frequency Likert Scale  Mean (± SD)  Median [IQR] | 3.41 (± 0.85)  3 [1] |
| How often are the patient’s real wishes followed during an EMS mission in a nursing home - Frequency Likert Scale  Mean (± SD)  Median [IQR] | 2.45 (± 0.66)  2 [1] |
| How often is a 112-call due to the unavailability of the general practitioner, nursing home physician or on call physician - Frequency Likert Scale  Mean (± SD)  Median [IQR] | 3.75 (± 0.78)  4 [1] |
| How do you assess the competence of the general practitioner provoking the 112-call - Adequacy Likert Scale  Mean (± SD)  Median [IQR] | 3.01 (± 0.87)  3 [2] |
| How strongly do you think the nursing home physician should be available 24/7 - Agreement Likert Scale  Mean (± SD)  Median [IQR] | 3.64 (± 1.24)  4 [2] |
| How strongly do you think hospitalizations should only be made after consultation with the general practitioner - Agreement Likert Scale  Mean (± SD)  Median [IQR] | 3.72 (± 1.21)  4 [2] |
| Which suggested interventions would seem useful to reduce the number of 112-calls in nursing homes, n (%)  More general practitioner interventions  Mobile geriatric nursing intervention teams  More nursing home staff  Better nursing home nurse education/competences  Mobile palliative care support teams  Telemedicine consultations  Specific EMS tiers designed for nursing home interventions  Other | 157 (82%)  100 (52%)  128 (67%)  148 (77%)  114 (65%)  38 (20%)  60 (31%)  26 (14%) |
| Which suggested interventions would seem useful to reduce the number of physician tier based EMS interventions in nursing homes, n (%)  More general practitioner interventions  Mobile geriatric nursing intervention teams  More nursing home staff  Better nursing home nurse education/competences  Mobile palliative care support teams  Telemedicine consultations  Specific EMS tiers designed for nursing home interventions  Other | 162 (84%)  91 (47%)  93 (48%)  132 (74%)  113 (59%)  42 (22%)  74 (38%)  18 (9.4%) |
| Which suggested interventions would seem useful to reduce the number of hospitalizations coming from nursing homes, n (%)  More general practitioner interventions  Mobile geriatric nursing intervention teams  More nursing home staff  Better nursing home nurse education/competences  Mobile palliative care support teams  Telemedicine consultations  Specific EMS tiers designed for nursing home interventions  Other | 146 (76%)  114 (59%)  126 (66%)  142 (74%)  130 (68%)  49 (26%)  37 (19%)  21 (11%) |
| Should teleconsultation play a bigger role in the care for chronic conditions - Agreement Likert Scale  Mean (± SD)  Median [IQR] | 3.32 (± 1.22)  4 [2] |
| Should teleconsultation play a bigger role in the care for acute conditions - Agreement Likert Scale  Mean (± SD)  Median [IQR] | 2.75 (± 1.12)  3 [2] |
| Are you interested in extra training in geriatrics or geriatric emergencies,  n (%)  Yes  No | 87 (45.3%)  104 (54.2%) |

EMS: Emergency Medical Services

**Supplementary Table S4**: Medical interventions

| Medical interventions | |
| --- | --- |
| How often are medical interventions performed by the EMS team (any member) during nursing home interventions - Frequency Likert Scale  Mean (± SD)  Median [IQR] | 3.08 (± 0.96)  3 [2] |
| How often are medical interventions performed by a physician during nursing home interventions - Frequency Likert Scale  Mean (± SD)  Median [IQR] | 2.65 (± 0.93)  3 [1] |
| How often could such interventions be avoided by more appropriate care by the nursing home - Frequency Likert Scale  Mean (± SD)  Median [IQR] | 3.89 (± 0.72)  4 [0] |
| Is a nurse allowed to decide on such medical interventions, n (%)  Yes  No | 73 (38%)  113 (58.9%) |
| Is a physician allowed to decide on such medical interventions, n (%)  Yes  No | 183 (95.3%)  9 (4.7%) |
| Do you think hospitalizations could be avoided if you had more authority concerning the initiation of such medical intervention, n (%)  Yes  No | 102 (53.1%)  81 (42.2%) |
| Do you think hospitalizations could be avoided if the nursing home staff had more authority concerning the initiation of such medical intervention, n (%)  Yes  No | 131 (68.2%)  61 (31.8%) |

EMS: Emergency Medical Services

**Supplementary Table S5**: Advance directives

| Advance directives | |
| --- | --- |
| Did you receive education on advance directives during your training, n (%)  Yes  No | 129 (67.2%)  62 (32.3%) |
| Are you legally bound to a negative advance directive if available, n (%)  Yes  No | 136 (70.8%)  55 (28.6%) |
| How often is there an advance directive available in the nursing home - Frequency Likert Scale  Mean (± SD)  Median [IQR] | 2.13 (± 0.66)  2 [0] |
| How often is a 112-call a call for advanced life support in spite of a negative advance directive - Frequency Likert Scale  Mean (± SD)  Median [IQR] | 3.55 (± 0.89)  4 [1] |
| Should a decision to transfer a nursing home resident with an advanced medical condition to the hospital be made more cautiously - Frequency Likert Scale  Mean (± SD)  Median [IQR] | 4.45 (± 0.74)  5 [1] |
| Should a decision to transfer a patient with advanced dementia to the hospital be made more cautiously - Frequency Likert Scale  Mean (± SD)  Median [IQR] | 4.51 (± 0.77)  5 [1] |
| How adequate is palliative care in nursing homes - Adequacy Likert Scale  Mean (± SD)  Median [IQR] | 2.17 (± 0.99)  2 [2] |
| How often are there un- or undertreated chronic conditions in nursing home residents during EMS interventions in nursing homes - Frequency Likert Scale  Mean (± SD)  Median [IQR] | 3.64 (± 0.88)  4 [1] |
| How often is there un- or undertreated chronic pain in nursing home residents during EMS interventions in nursing homes - Frequency Likert Scale  Mean (± SD)  Median [IQR] | 3.19 (± 0.81)  3 [1] |

EMS: Emergency Medical Services

**Supplementary Table S6**: Emotions

| Emotions | |
| --- | --- |
| Are you afraid of the medical implications if you do too little - Frequency Likert Scale  Mean (± SD)  Median [IQR] | 2.75 (± 1.21)  3 [2] |
| Are you afraid of the medical implications if you do too much - Frequency Likert Scale  Mean (± SD)  Median [IQR] | 2.39 (± 1.21)  2 [1] |
| How often does the 112-call shift the problem away from the nursing home personnel towards someone else - Frequency Likert Scale  Mean (± SD)  Median [IQR] | 3.98 (± 0.86)  4 [1] |
| How often does hospitalization shift the problem away from the EMS team towards someone else - Frequency Likert Scale  Mean (± SD)  Median [IQR] | 3.83 (± 0.86)  4 [1] |
| How do you feel when you hospitalize a nursing home resident, n (%)  Angry  Disappointed  Scared  Stressed  Frustrated  Satisfied  Relieved  Happy  Neutral  Other | 18 (10%)  95 (51%)  4 (2.1%)  1 (0.5%)  115 (61%)  22 (12%)  8 (4.3%)  2 (1.1%)  3 (1.6%)  39 (21%) |

EMS: Emergency Medical Services

**Supplementary Table S7**: Comparison between nurses and physicians regarding the general characteristics of EMS interventions in nursing homes

| Characteristics of EMS interventions in nursing homes (general information) | | | |
| --- | --- | --- | --- |
| *Variable* | *Nurse*  *(n = 78)* | *Physician*  *(n = 114)* | *p-value* |
| Is there adequately staffed personnel in nursing homes in numbers, n (%)  Yes  No | 3 (3.8%)  75 (96.2%) | 13 (11.4%)  98 (86%) | 0.056 |
| Is there adequately staffed personnel in nursing homes in competence, n (%)  Yes  No | 1 (1.3%)  76 (97.4%) | 6 (5.3%)  107 (93.9%) | 0.342 |
| Is there a difference in staffing between nights and weekends, n (%)  Yes  No | 75 (96.2%)  3 (3.8%) | 111 (97.4%)  2 (1.8%) | 0.480 |
| Are there more EMS interventions during weekends and nights, n (%)  Yes  No | 53 (67.9%)  24 (30.8) | 65 (57%)  48 (42.1%) | 0.278 |
| Does the difference in staffing impact the quality of care, n (%)  Yes  Sometimes  No | 59 (75.6%)  17 (21.8%)  0 (0%) | 71 (62.3%)  38 (33.3%)  2 (1.8%) | 0.192 |

EMS: Emergency Medical Services

**Supplementary Table S8:** Comparison between nurses and physicians regarding the specific characteristics of EMS interventions in nursing homes

| Characteristics of EMS interventions in nursing homes (specific information) | | | |
| --- | --- | --- | --- |
| *Variable* | *Nurse*  *(n = 78)* | *Physician*  *(n = 114)* | *p-value* |
| How often is there telephone contact with the general practitioner before the initiation of a 112-call? n (%)  1: Never  2: Almost never  3: Sometimes  4: Often  5: Almost always  Median [IQR] | 7 (9.9%)  22 (28.2%)  29 (37.2%)  18 (23.1%)  2 (2.6%)  3 [2] | 12 (10.5%)  44 (39.2%)  40 (35.1%)  16 (14%)  2 (1.8%)  3 [1] | 0.419      0.073 |
| How often is the general practitioner present before initiation of a 112-call? n (%)  1: Never  2: Almost never  3: Sometimes  4: Often  5: Almost always  Median [IQR] | 12 (15.4%)  43 (55.1%)  19 (24.4%)  4 (5.1%)  0 (0%)  2 [1] | 18 (15.9%)  76 (67.3%)  17 (15%)  2 (1.8%)  0 (0%)  2 [0] | 0.0181    0.120 |
| How often is the placement of a 112-call in accordance with the patient’s wishes? (Fig. 1) n (%)  1: Never  2: Almost never  3: Sometimes  4: Often  5: Almost always  Median [IQR] | 13 (19.7%)  46 (59%)  15 (19.2%)  4 (5.1%)  0 (0%)  2 [1] | 9 (8%)  58 (51.8%)  45 (40.2%)  0 (0%)  0 (0%)  2 [1] | 0.001      0.021 |
| How often is a 112-call due to an acute emergency? (Fig. 2) n (%)  1: Never  2: Almost never  3: Sometimes  4: Often  5: Almost always  Median [IQR] | 6 (7.7%)  45 (57.7%)  22 (28.2%)  5 (6.4%)  0 (0%)  2 [1] | 6 (5.3%)  52 (46%)  38 (33.6%)  14 (12.4%)  3 (2.7%)  2 [1] | 0.213      0.031 |
| How often are EMS interventions in nursing homes really necessary or indicated? n (%)  1: Never  2: Almost never  3: Sometimes  4: Often  5: Almost always  Median [IQR] | 2 (4.9%)  52 (51.3%)  22 (18.9%)  2 (2.9%)  0 (0%)  2 [1] | 10 (7.1%)  73 (65.2%)  24 (27.1%)  5 (4.1%)  0 (0%)  2 [1] | 0.232      0.237 |
| How often is the appropriate EMS level activated? n (%)  1: Never  2: Almost never  3: Sometimes  4: Often  5: Almost always  Median [IQR] | 5 (6.4%)  39 (50%)  25 (32.1%)  7 (9%)  2 (2.6%)  2 [0] | 6 (5.3%)  66 (57.9)  33 (29.9%)  8 (7%)  1 (0.9%)  2 [1] | 0.763    0.390 |
| How often is a EMS team with nurse (PIT) unjustly activated? (Fig 3) n (%)  1: Never  2: Almost never  3: Sometimes  4: Often  5: Almost always  Median [IQR] | 2 (2.6%)  12 (15.4%)  24 (30.8%)  36 (46.2%)  4 (5.1%)  2 [1] | 2 (1.9%)  19 (17.8%)  56 (52.3%)  29 (27.1%)  1 (0.9%)  3 [1] | 0.013      0.010 |
| How often is a EMS team with physician (MUG) unjustly activated? n (%)  1: Never  2: Almost never  3: Sometimes  4: Often  5: Almost always  Median [IQR] | 1 (1.3%)  8 (10.4%)  9 (11.7%)  43 (55.8%)  16 (20.8%)  4 [1] | 1 (0.9%)  6 (5.4%)  16 (14.4%)  62 (55.9%)  26 (23.4%)  4 [1] | 0.739    0.514 |
| How often are hospitalizations avoidable? n (%)  1: Never  2: Almost never  3: Sometimes  4: Often  5: Almost always  Median [IQR] | 3 (1.2%)  6 (7.7%)  32 (41%)  29 (37.2%)  8 (10.3%)  3 [1] | 0 (0%)  13 (11.4%)  53 (46.5%)  38 (33.3%)  5 (8.8%)  3 [1] | 0.227      0.582 |
| How often are the patient’s real wishes followed during an EMS mission in a nursing home? (Fig 4) n (%)  1: Never  2: Almost never  3: Sometimes  4: Often  5: Almost always  Median [IQR] | 5 (6.4%)  52 (66.7%)  16 (20.5%)  5 (6.4%)  0 (0%)  3 [1] | 3 (2.6%)  48 (42.1%)  58 (50.9%)  5 (4.4%)  0 (0%)  3 [1] | < 0.001      < 0.001 |
| How often is a 112-call due to the unavailability of the general practitioner. nursing home physician or on call physician? n (%)  1: Never  2: Almost never  3: Sometimes  4: Often  5: Almost always  Median [IQR] | 1 (0.4%)  4 (5.2%)  19 (24.7%)  43 (5.8%)  10 (13%)  4 [1] | 0 (0%)  7 (6.2%)  28 (24.8%)  63 (55.8%)  15 (13.3%)  4 [1] | 0.818    0.945 |
| How do you assess the competence of the general practitioner provoking the 112-call? n (%)  1: Very inadequate  2: Inadequate  3: Neutral  4: Adequate  5: Very adequate  Median [IQR] | 4 (5.1%)  17 (21.8%)  43 (55.1%)  13 (16.7%)  1 (1.3%)  3 [1] | 3 (2.7%)  27 (23.9%)  43 (38.1%)  35 (31%)  5 (4.4%)  3 [2] | 0.060      0.079 |
| How strongly do you think the nursing home physician should be available 24/7? (Fig 5) n (%)  1: I do not agree at all  2: I do not agree  3: Neutral  4: I agree  5: I fully agree  Median [IQR] | 0 (0%)  8 (10.3%)  13 (16.7%)  26 (33.3%)  31 (39.7%)  4 [2] | 14 (12.3%)  17 (14.9%)  23 (20.2%)  33 (28.9%)  27 (23.7%)  4 [2] | 0.005      < 0.001 |
| How strongly do you think hospitalizations should only be made after consultation with the general practitioner? n (%)  1: I do not agree at all  2: I do not agree  3: Neutral  4: I agree  5: I fully agree  Median [IQR] | 3 (3.9%)  15 (19.5%)  16 (20.8%)  20 (26%)  23 (26.3%)  4 [2] | 5 (4.4%)  16 (14.2%)  17 (15%)  33 (29.2%)  42 (37.2%)  4 [2] | 0.631      0.190 |

EMS: Emergency Medical Services; PIT: Paramedic Intervention Team; MUG: Mobile Urgency Group

**Supplementary Table S9:** Comparison between nurses and physicians regarding medical interventions

| Medical interventions | | | |
| --- | --- | --- | --- |
| *Variable* | *Nurse*  *(n = 78)* | *Physician*  *(n = 114)* | *p-value* |
| How often are medical interventions performed by the EMS team (any member) during nursing home interventions? n (%)  1: Never  2: Almost never  3: Sometimes  4: Often  5: Almost always  Median [IQR] | 1 (1.3%)  18 (23.1%)  29 (37.2%)  26 (33.3%)  4 (5.1%)  3 [1] | 3 (2.6%) 36 (31.6%) 42 (36.8%) 22 (19.3%) 11 (9.6%)  3 [2] | 0.168    0.166 |
| How often are medical interventions performed by a physician during nursing home interventions? n (%)  1: Never  2: Almost never  3: Sometimes  4: Often  5: Almost always  Median [IQR] | 6 (7.7%)  31 (39.7%)  28 (35.9%)  12 (15.4%)  1 (1.3%)  3 [1] | 8 (7%)  50 (43.9%)  33 (28.9%)  19 (16.7%)  4 (3.5%)  2 [1] | 0.764    0.989 |
| How often could such interventions be avoided by more appropriate care by the nursing home? n (%)  1: Never  2: Almost never  3: Sometimes  4: Often  5: Almost always  Median [IQR] | 0 (0%)  1 (1.3%)  13 (16.7%)  48 (62.8%)  15 (19.2%)  4 [0] | 0 (0%)  6 (5.3%)  27 (23.7%)  63 (55.3%)  18 (15.8%)  4 [1] | 0.275    0.104 |
| Is a nurse allowed to decide on such medical interventions, n (%)  Yes  No | 28 (35.9%)  50 (64.1%) | 45 (39.5%)  63 (55.3%) | 0.087 |
| Is a physician allowed to decide on such medical interventions. n (%)  Yes  No | 73 (93.6%)  5 (6.4%) | 110 (96.5%)  4 (3.5%) | 0.350 |
| Do you think hospitalizations could be avoided if you had more authority concerning the initiation of such medical intervention, n (%)  Yes  No | 57 (73.1%)  21 (26.9%) | 45 (39.5%)  60 (52.6%) | < 0.001 |
| Do you think hospitalizations could be avoided if the nursing home staff had more authority concerning the initiation of such medical intervention, n (%)  Yes  No | 54 (69.2%)  24 (30.8%) | 77 (67.5%)  37 (32.5%) | 0.805 |

EMS: Emergency Medical Services

**Supplementary Table S10:** Comparison between nurses and physicians regarding advance directives

| *Advance directives* | | | |
| --- | --- | --- | --- |
| *Variable* | *Nurse*  *(n = 78)* | *Physician*  *(n = 114)* | *p-value* |
| Did you receive education on advance directives during your training, n (%)  Yes  No | 56 (71.8%)  22 (28.2%) | 73 (64%)  40 (35.1%) | 0.411 |
| Are you legally bound to a negative advance directive if available, n (%)  Yes  No | 62 (79.5%)  16 (20.5%) | 74 (64.9%)  39 (34.2%) | 0.078 |
| How often is there an advance directive available in the nursing home? n (%)  1: Never  2: Almost never  3: Sometimes  4: Often  5: Almost always  Median [IQR] | 12 (15.4%)  50 (64.1%)  12 (15.4%)  4 (5.1%)  0 (0%)  2 [0] | 12 (10.6%)  75 (66.4%)  23 (20.4%)  3 (2.7%)  0 (0%)  2 [0] | 0.520    0.464 |
| How often is a 112-call a call for advanced life support in spite of a negative advance directive? n (%)  1: Never  2: Almost never  3: Sometimes  4: Often  5: Almost always  Median [IQR] | 0 (0%)  4 (5.1%)  24 (30.8%)  36 (46.2%)  14 (17.9%)  4 [1] | 4 (3.5%)  14 (12.4%)  35 (31%)  52 (46%)  8 (7.1%)  4 [1] | 0.039      0.014 |
| Should a decision to transfer a patient with an advanced medical condition to the hospital be made more cautiously? n (%)  1: Never  2: Almost never  3: Sometimes  4: Often  5: Almost always  Median [IQR] | 0 (0%)  1 (1.3%)  4 (5.1%)  32 (41%)  41 (52.6%)  4.5 [1] | 2 (1.8%)  1 (0.9%)  7 (6.1%)  37 (32.5%)  67 (58.8%)  5 [1] | 0.594    0.538 |
| Should a decision to transfer a patient with advanced dementia to the hospital be made more cautiously?  n (%)  1: Never  2: Almost never  3: Sometimes  4: Often  5: Almost always  Median [IQR] | 1 (1.3%)  2 (2.6%)  5 (6.4%)  26 (33.3%)  44 (56.4%)  5 [1] | 1 (0.9%)  2 (1.8%)  4 (3.6%)  28 (25.2%)  5 (68.5%)  5 [1] | 0.550      0.085 |
| How adequate is palliative care in nursing homes?  n (%)  1: Very inadequate  2: Inadequate  3: Neutral  4: Adequate  5: Very adequate  Median [IQR] | 21 (26.9%)  31 (33.5%)  19 (24.4%)  5 (6.4%)  2 (2.6%)  2 [2] | 29 (25.7%)  51 (45.1%)  21 (18.6%)  9 (8%)  3 (2.7%)  2 [2] | 0.873      0.872 |
| How often are there un- or undertreated chronic conditions in nursing home residents during EMS interventions in nursing homes? n (%)  1: Never  2: Almost never  3: Sometimes  4: Often  5: Almost always  Median [IQR] | 0 (0%)  4 (5.1%)  26 (33.3%)  33 (42.3%)  15 (19.2%)  4 [1] | 0 (0%)  15 (13.3%)  38 (33.6%)  42 (37.2%)  18 (15.9%)  4 [1] | 0.298    0.152 |
| How often is there un- or undertreated chronic pain in nursing home residents during EMS interventions in nursing homes? n (%)  1: Never  2: Almost never  3: Sometimes  4: Often  5: Almost always  Median [IQR] | 1 (1.3%)  7 (9%)  36 (46.2%)  28 (35.9%)  6 (7.7%)  3 [1] | 1 (1.2%)  28 (24.8%)  49 (43.4%)  34 (30.1%)  1 (0.9%)  3 [2] | 0.013      0.006 |

EMS: Emergency Medical Services

**Supplementary Table S11:** Comparison between nurses and physicians regarding emotions

| Emotions | | | |
| --- | --- | --- | --- |
| *Variable* | *Nurse*  *(n = 78)* | *Physician*  *(n = 114)* | *p-value* |
| Are you afraid of the medical implications if you do too little? n (%)  1: Never  2: Almost never  3: Sometimes  4: Often  5: Almost always  Median [IQR] | 6 (7.7%)  22 (28.2%)  27 (34.6%)  14 (17.9%)  9 (11.5%)  3 [2] | 30 (26.3%)  27 (23.7%)  20 (17.5%)  33 (28.9%)  4 (3.5%)  3 [3] | 0.001    0.048 |
| Are you afraid of the medical implications if you do too much? n (%)  1: Never  2: Almost never  3: Sometimes  4: Often  5: Almost always  Median [IQR] | 10 (12.8%)  28 (35.9%)  28 (35.9%)  6 (7.7%)  6 (7.7%)  2.5 [1] | 38 (33.3%)  34 (29.8%)  21 (18.4%)  19 (16.7%)  2 (1.8%)  2 [2] | 0.001    0.015 |
| How often does the 112-call shift the problem away from the nursing home personnel towards someone else? n (%)  1: Never  2: Almost never  3: Sometimes  4: Often  5: Almost always  Median [IQR] | 1 (1.3%)  2 (2.6%)  13 (16.7%)  39 (50%)  23 (29.5%)  4 [1] | 3 (2.6%)  3 (2.6%)  21 (18.4%)  58 (50.9%)  29 (25.4%)  4 [1] | 0.942      0.470 |
| How often does the 112-call shift the problem away from the EMS team towards someone else? n (%)  1: Never  2: Almost never  3: Sometimes  4: Often  5: Almost always  Median [IQR] | 3 (3.8%)  3 (3.8%)  23 (29.5%)  32 (41%)  17 (21.8%)  4 [1] | 3 (2.6%)  6 (5.3%)  19 (16.7%)  57 (50%)  29 (25.4%)  4 [2] | 0.292    0.159 |

**Supplementary Table S12a:** Open questions (the answers have not been modified from their original version)

| **How would you define an avoidable hospitalization?** |
| --- |
| Tijdige interventie door huisarts, eigenlijke wens patient of familie was in home te blijven |
| Vroegtijdige zorgplanning die niet gerespecteerd wordt |
| Door een sneller contact met huisarts te nemen kunnen er sneller therapiën uitgevoerd worden. Dmv vroegtijdige zorgplanning kan er beslist worden om niet meer over te gaan tot een ziekenhuisopname. |
| Vroegtijdige zorgplanning en DNR registratie. Correct inschakelen HA |
| Ietwat intensievere medische verzorging ter plaatse is meestal niet mogelijk wegens personeelsgebrek |
| Chronische aandoening met DNR code wordt nog naar ZH gebracht, opname kort voor weekend of feestdag gezien eigen huisarts niet van wacht |
| Een opname die het gevolg is van niet correct medisch handelen uren/dagen voorafgaande aan de oproep en/of het niet eerbiedigen van een voorafbestaande wils/zorgbeschikking. |
| Sneller contact met de huisarts en / of specialist zodat er tijdens de daguren nog iets kan opgelost of voorzien worden. |
| Onnuttige oproepen, patiënten die geen ziekenhuisopname meer wensen, DNR 3 codes |
| End of life pathologie, die door de HA ter plaatste uitgevoerd kan worden. |
| negatieve wilsverklaring of palliatieve setting |
| Sociale opname |
| Bij een goede wilsverklaring en een duidelijk plan van aanpak bij patiënten, is een opname vaak te vermijden. |
| comfortzorg in WZC |
| Dit is een opname die eerder via een dagopname/consultatie kan mbt algemene toestand. |
| Ten koste van het comfort van de patiënt, geen dnr code aanwezig |
| Indien opvolging via het personeel en bezoek via de huisarts volstaat. Bijkomend onbreken van een (realistische) wilsverklaring. |
| Een opname waarbij de graad en / of intensiteit van zorg dezelfde is als deze redelijkerwijze in het woonzorgcentrum zou uitgevoerd kunnen worden (indien adequate bestaffing aanwezig zou zijn). |
| palliatieve setting, en inzicht van de familie in de uitzichtloosheid |
| comforttherapie, deftige opvolging huisarts/RVTarts |
| Betere zorgplannen, huisarts die niet komt kijken |
| Geen meerwaarde van ziekenhuisopname op uitkomst of comfort patient |
| Patiënten die dnr code hebben en huisarts die zelf ter plaatse komt |
| Geen toegevoegde waarde voor levenskwaliteit patiënt of vermijdbaar door voorafgaand uitgebreid gesprek HA met patiënt/familie rond therapiebeperking |
| HuisArts ter plaatse laten komen voor nazicht |
| Palliatieve zorg |
| Opname die had vermeden kunnen worden indien er vroeger in het zorgplan van de patiënt correcte medische hulp is ingeroepen of concrete wilsverklaringen werden opgesteld. |
| algemene achteruitgang, te weinig personeel in de weekends in de WZC |
| Correcte palliatieve zorg in wzc opgestart |
| Enkel kleine wijzigingen aan medicatie noodzakelijk alvorens ze onmiddellijk naar het ziekenhuis te rijden. Koorts betekent niet dat er onmiddellijk naar een ziekenhuis moet worden gegaan… |
| Ter plaatse adequate medicatie, voldoende VPK-verzorgenden om de juiste zorg te kunnen toedienen. Adequaten kennis van pten en hun te verwachten ziektebeeld. |
| Door beter af te stemmen met de huisarts hoe te reageren bij achteruitgang of opkomen van een bepaald probleem. Door niet af te wachten tot 's avonds, tot de kantooruren gedaan zijn en disciplines niet meer telefonisch bereikbaar zijn. Veel problemen kunnen via de poli opgelost worden en hoeven de 112 centrale niet te passeren. |
| Wanneer op voorhand voor alle partijen een heel duidelijk DNR-beleid gekend is en deze ook effectief wordt nagevolgd |
| Patiënt die ambulant door huisarts en verpleegkundige kan opgevolgd worden |
| Betere DNR codering voor bewoners WZC, snellere detectie van AAT, betere opvolging van status patiënt middels meer personeel en vnl meer verpleegkundig personeel |
| Indien aanwezigheid van voldoende en correct opgeleide verpleegkundigen en dus bekwame vpk zou de patient bijna altijd in het rvt moeten kunnen blijven. Ik denk dan bvb aan het geven van goede pijnstilling, infuustherapie, opzet van een voornaam palliatief team, preventie van vallen, vroegtijdig herkennen van zieker wordende patiënt en daar tijdig op inspelen bij voorkeur door de continue aanwezigheid van een (opnieuw bekwame) huisarts. Maw een soort ziekenhuisomgeving creeeren in een wzc en verplichte opmaak van een DNR code van zodra patient niet meer reintegreerbaar is in de maatschappij (dus bij chronisch verblijf met oog op levenseinde en dus niet van toepassing op kortverblijf). Samen met goede en duidelijke afspraken richting patient en familie. |
| algemene achteruitgang. |
| palliatieve setting |
| Wanneer een patiënt dit niet wil |
| meestal chronische pathologie |
| gekende palliatieve toestand |
| Situaties waarin de huisarts ook een behandeling kan opstarten |
| Indien er een tijdige en adequate interventie gebeurd zou zijn en de patiënt dus tijdig behandeld was in het WZC dan kan een ziekenhuisopname vermeden worden bijvoorbeeld vermoeden van een urineweginfectie sneller een staalname door verpleegkundige in het WZC en tijdig contact opnemen met de huisarts in plaats van onnodig lang wachten. |
| palliatieve settings, chronische ziektes, DNR |
| Wanneer de situatie palliatief is en deze comfortzorgen in het rusthuis kunnen worden uitgevoerd, beter beleid hierrond is noodzakelijk |
| realistische verwachtingen hebbern |
| Onbekwame beoordeling van de ernst. |
| patienten die aan het einde van hun leven zijn, hebben geen meerwaarde bij trfrt naar ziekenhuis |
| Ziekenhuisopname die niet nodig was geweest na duidelijke communicatie naar de familie en patiënt toe om de verwachtingen omtrent behandeling en ziekenhuisopname te kaderen. Vaak is er een vraag naar ziekenhuisopname met onrealistische verwachtingen naar behandeling en prognose van de patiënt toe. Therapiebegrenzing en lange termijnsprognose van bepaalde aandoeningen zijn zelden tot nooit in detail doorgesproken met familie en patiënt (vb. de gevolgen van een reanimatie, beademing, dialyse, ... lange termijnsprognose van dementie, kanker, overleving na heupfracturen,...) met alle gevolgen van dien. |
| beter communicatie tussen huisarts/patient/familie/verpleging |
| uitzichtloze situatie met verwachte deterioratie en planbare DNR en palliatief verloop |
| te laat ingegrepen |
| deftig DNR beleid concrete afspraken met pt en naaste |
| Opvolging huisarts |
| Een opname is vermijdbaar indien dezelfde zorg ook door verpleegkundig personeel in het woonzorgcentrum kan uitgevoerd worden, onder begeleiding van huisarts. |
| Chronisch probleem, dat in goed overleg met de huisarts en het personeel in het wzc kan opgevangen worden. Overlijden van een bewoner waar een mug-arts bij komt is compleet zinloos. |
| DNR 2 of 3 coderingen, reeds langer aanslepend probleem dat niet tijdig werd opgemerkt, Stervende patiënt die slechts enkele uren nog te leven heeft. |
| zorg die door verpleegkundige/zorgkundige in het woonzorgcentrum kan uitgevoerd worden, en kan opgevolgd worden door de huisarts. |
| Betere zorg in WZC, huisarts eerst te contacteren, interventies ter plaatse |
| ziekenhuisopname wegens ontoereikende eerstelijnszorg/adekwate huisartsgeneeskunde |
| Daar waarbij er eigenlijk al een wilsverklaring is van patiënt of familie of er één zou moeten zijn en deze niet gerespecteerd wordt door huisarts of WZC personeel |
| Opname waarbij niet geluisterd wordt naar de wensen van de patiënt, progressie aandoening waarbij onvoldoende geanticipeerd werd, onduidelijke therapiebeperkingscode |
| Voldoende en adequate zorg |
| Een pat die DNR is, kan in het WZC blijven en hoeft niet naar het ziekenhuis |
| Futiele zorg |
| Op vraag van familie, acute decompensatie van chronische aandoening of pijnproblematiek( die kan aangepakt worden door HA ) |
| Duidelijke afspraken rond therapiebeperking. |
| Opnames tgv niet correct en snel opvolgen en onvoldoende kennis over de aanwezige patiënten |
| Opname met een negatieve wilsbeschikking |
| geen acuut probleem of reeds duidelijk DNR beleid afgetekend |
| Snel reversiebele oorzaak - DNR 2 |
| Opname waarbij mits voorafgaand goed afgesproken zorgplanning even goede tot soms betere zorg in het WZC kan voorzien worden |
| Een opname die door snellere detectie en interventie niet moet plaatsvinden |
| Planbare zorg |
| DNR code bespreken met pt en familie en optimaal behandelen in rvt zonder nog behandeling in hospitaal te starten |
| Door grondig nazicht |
| Indien adequate DNR codering |
| Behandeling door huisarts mogelijk/ wanneer patiënt reeds therapie beperking heeft maar hier niet naar gekeken wordt |
| Niet uitgevoerd indien vroegtijdige zorgplanning besproken zou zijn |
| Ziekenhuisopname die vermijdbaar zou zijn indien de huisarts beschikbaar was of indien er op voorhand al een therapeutisch plan (moet/wil deze patiënt nog naar het ziekenhuis voor zorgen) gemaakt zou zijn |
| enerzijds voldoende snelle interventie van vpk en ha zodat geen escalatie van zorg nodig is, anderzijds meer discussie over levenskwaliteit waardoor minder zinloze zorg wordt geleverd |
| Een opname waarbij geen bijkomende therapie gestart wordt, of noodzakelijke investigaties ter evaluatie van mogelijk behandelbare diagnoses uitgevoerd worden, behalve comforttherapie die ook in het woonzorgcentrum gestart zou kunnen worden. |
| Huisartsopvolging |
| Slechte EWS voorafgaandelijk aan activatie |
| prehospitaal heeft geen zin ,mensen zijn soms te ver gevorderd of te oud |
| Betere zorg pre-hospitaal en inschatting met behandeling |
| ui!tgesproken en correct zorgplan op voorhand opgesteld, zoals correct DNR beleid, ook besproken met familie. |
| Voorafbestaande wilsbeschikking, terminale patiënten, uitzichtloze situatie bij bvb recidiverende opnames |
| Als de nodige zorg ook buiten de ziekenhuissetting kan toegediend worden is de opname vermijdbaar. |
| Therapiebeperking met achteruitgang |
| Wanneer via wilsverklaring beschreven staat geen ziekenhuisopnames meer; tijdig HA verwittigen; Therapie afwachten gedurende >24u |
| Door meer personeel in een RVT en meer bezoeken van de huisartsen zouden er meer zorgen kunnen uitgevoerd worden in het RVT zelf ipv een onnodige tranfer uit te voeren |
| Comfortzorg in rvt |
| Kennis correcte wilsbeschikking patiënt, voldoende opleiding personeel om alarmsignalen te detecteren bij een patiënt |
| Palliatieve setting: te vermijden door goede zorgplanning en communicatie met pt, familie en zorgpersoneel |
| Betere opvolging huisartsen, beter bespreken DNRcodering, duidelijkheid van deze codering |
| Eerst gezien worden door arts (die pat. kent) ipv 'telefonisch' door te verwijzen |
| Pt voelt zich goed en wenst liever in zijn vertrouwde omgeving te verblijven. |
| Niet enkel telefonisch advies maar ook ter plaatse komen om de patient te zien |
| bij correct einde levensbeleid, correcte behandeling en opvolging, voldoende zorgomkadering, val preventie,... Een definitie is niet eenvoudig, het is een veelvoud aan factoren. |
| futiele zorg - vergaande zorgvraag (vb. intubatie, IZ) bij patiënten met belangrijk cognitief deficiet.  niet respecteren DNR |
| Genoeg comfort en zorg voor de bewoner in het rusthuis |
| Voorafgaande DNR codering en houden aan vooraf gemaakte afspraken. |
| respecteren DNR codering, zorg door prehospitaal team cfr. PHYSICIAN RESPONSE UNIT (PRU) |
| Een opname die geen verbeterende invloed heeft op de levenskwaliteit over een termijn van bijvoorbeeld een jaar. |
| Een vermijdbare ziekenhuisopname is een opname die technisch, logistiek en farmacologisch perfect mogelijk is in het woonzorgcentrum indien goed omkaderd. |
| Palliatief beleid |
| soms opnames van personen terwijl uitdrukkelijk vermeld staat dat ze geen opname meer wensen. Soms kan een bezoek van de huisarts ter plaatse al veel oplossen. |
| Een opname die mits eerdere adequate behandeling in het woonzorgcentrum vermeden had kunnen worden |
| Patiënt wenst geen opname of interventies meer, dus comfortzorg primeert - preventieve of ambulante zorg zou volstaan hebben. |
| situatie waarbij vroegtijdig zou geanticipeerd zijn op het ziektebeeld dat patiënt stelde en/of bepaling van vroegtijdige zorgplanning waarbij onnodige transfer naar ziekenhuis kan vermeden worden |
| tijdig consult van de waarnemende arts bij optreden van symptomen , verantwoordelijke arts die patient ziet en overleg pleegt met familie , durft medische beslissingen te nemen |
| indien huisarts zou langsgeweest zijn kan een opname vermeden worden |
| Opvolging door huisarts mogelijk |
| Het voorkomen van een ziekenhuisopname. |
| Therapeutische hardnekkigheid.  DNR codes niet respecteren. |
| Opname voor een chronisch deteriorende patiënt, waarbij in het WZC niet , of niet duidelijk genoeg afspraken zijn gemaakt met patiënt of met name de familie rond planning van zorg. Vaak patiënten die zich nog niet lang in het WZC bevinden, of die recent naar het ziekenhuis geweest zijn. Meestal in de nacht, als men de patiënt ook niet goed kent. Dus vaak nog niet zo goed gekende patiënten door de verpleging/verzorgend personeel. |
| Patiënt met uitgebreid DNR beleid die door infrastructurele en personele tekorten niet adequaat kunnen verzorgt worden in het rusthuis en waarbij transfer naar het ziekenhuis enkel resulteert in comfortzorgen |
| Goed gedocumenteerde wens patient |
| Door betere preventie en medische expertise te implementeren zouden er vele ziekenhuisopnames vermeden kunnen worden en in het woonzorgcentrum behandeld kunnen worden. |
| Patiënt die behandeling daar zou kunnen krijgen mits genoeg competente personeel. Freiwent voorbeeld : palliatieve/ terminale situaties |
| Een ziekenhuisopname van dermate milde pathologie dat ze ook door de huisarts kan opgevolgd worden, of een dergelijke infauste prognose dat overlijden binnen de paar uur verwacht kan worden en palliatief thuisbeleid kan opgestart worden. |
| Noodzaak tot onderzoek, opname conform wens patiënt en therapiebeperkingen. |
| - comfortzorg  - consultatie die kan gebeuren door huisarts  - consultatie die kan gebeuren op raadpleging in her ziekenhuis |
| (Her)Plaatsen van sonde, dossier zegt geen ziekenhuiopname, overleg met familie |
| Situatie waarin een ziekenhuisopname geen enkele extra meerwaarde kan bieden voor de patiënt t.o.v. zorgen die ook kunnen worden opgestart vanuit het rusthuis. |
| Opname die vermeden kon worden indien huisarts reeds ter plaatse gegaan zou zijn of indien er voorheen duidelijk afspraken met patiënt of familie werden gemaakt. Nood aan voldoende omkadering en kennis bij personal zodat comforttherapie in woonzorgcentrum ook opgestart kan worden. |
| Pt heeft al DNR en voldoet niet aan criteria opname; pt maakte een mineure val door maar vertoont geen tekenen van contusie, breuk of cerebrale hemorrhagie, terwijl de MUG gebeld werd met vraag naar monitoring |
| Weinig uitzicht op betere levenskwaliteit bij ziekenhuisopname |
| meer overleg over DNR-codes, meer medicatie in rusthuizen, meer realistische prognoses opstellen -- mi gaat het wel meer over vermijdbare mug-oproepen dan over vermijdbare ZHopnames |
| achteruitgang patient met dnr code die liefst in wzc zou komen te overlijden, familie wil dit niet of personeel kan dit niet. |
| Een vermijdbare ziekenhuisopname is wanneer een patiënt naar het ziekenhuis gebracht wordt waarbij er interventies gestelt worden die ambulant konden plaatsvinden. |
| Mensen die stervende zijn. |
| Opstarten van aduequate therapie in wzc, met gecompetenteerd en genoeg verpleegkundig personeel |
| Een oproep waarbij je de situatie ter plaatse kunt oplossen bvb hypoglycemie oplossen. |
| Betere opvolging door HA |
| Een chronische aandoening die al maanden bezig is, maar vrijdag avond plots toeneemt ? |
| Geen meerwaarde voor opname in het ziekenhuis |
| MUG interventies in RVT per definitie geen goede zorg denk ik, huisarts ter plaatse, proberen problemen op te lossen, bij levensbedreigend acuut event, eerder degelijke DNR voorzien... |
| Minimaal alvast Dnr codering - doch: is opvang ter plekke wel kwalitatief, humaan? |
| Door tijdig planbare zorg en beslissingen rondom levenseinde |
| Een niet acuut probleem dat met de middelen beschikbaar in het ambulant milieu (verpleegkundige aan huis, huisarts, middelen van het RVT) naar behoren kunnen worden aangepakt. |
| Een pt die niet naar ziekenhuis zou moeten als de situatie op tijd correct zou ingeschat zijn door verplegend personeel en huisarts, of wanneer er niet gesproken is over wilsbeschikking of zorgplanning. |
| Slecht gedetineerde DNR beleiden en therapeutische hardnekkigheid bij infauste prognose |
| Patiënt met een DNR 3 beleid. Patiënt met een aanslepend medisch probleem zonder adequate opstart van therapie via de huisarts. |
| als beterschap bekomen had kunnen worden met tijdige en adequate zorg en herkenning |
| dit is een ziekenhuisopname die kan vermeden worden als er een duidelijk medisch dossier, duidelijke DNR code, duidelijke vroegtijdige zorgplanning aanwezig is zodat dit kan gevolgd worden door het personeel in het WZC iom de huisarts. |
| indien er duidelijk een lange aanloop bij de patiënt terug te vinden is en er gewoon geen adequate actie ondernomen is, toen het nog om iets mineurs ging; of wanneer een familielid beslist om in acute setting tegen de uitgebreide therapiebeperking in te gaan |
| minder menselijke zorg, geen medische meerwaarde |
| Opnames wegens trage achteruitgang tot onhoudbare situatie die met vroege interventies en goede opvolging van huisarts vermeden hadden kunnen worden. |
| Palliative setting, algemene achteruitgang zonder acute detoriatie, vermoeden heup# bij dementerende bedlederige fraile patiënt waarbij toch geen chirurgisch ingrijpen meer zal gebeuren |
| Une prise en charge débouchant sur un retour ou un maintient de la personne âgée en maison de repos moyennant une discussion préalable avec le médecin traitant le patient et éventuellement la famille avec si nécessaire une modification de la surveillance locale du patient et si nécessaire de son traitement |
| Fin de vie, projet de vie |
| Souhaits de fins de vie ou pathologies chroniques non curables mal traités/connues en mrs |
| Soins appropriés en MRS |
| Prise en charge sur place possible |
| un plan de soins ,sur la fin de vie clair établi et revu régulièrement lors du séjour |
| tout dépend ce qu'évitable veut dire : non urgente ? injustifiée ? La plupart du temps, il n'y a pas d'événement hyperaigu nécessitant une intervention médicale immédiate et des soins hospitaliers immédiats garantissant la survie, l'autonomie , la qualité de vie et la dignité du patient. MAiS le système de santé belge n'est pas prêt à prendre en considération que la mort fait partie du cycle de la vie et peut normalement survenir en structure de soins. |
| hospitalisation futile ou rendue nécessaire par manque de soins sur place |
| hospi qui n'améliore ni l'état de santé ni la qualité de vie du patient |
| une hospitalisation non urgente, qui pourrait être programmée, ou un soin/traitement qui pourrait être réalisé par le personnel de la MR si consignes claires du médecin généraliste |
| majoration des soins en maison de repos permettant au patient de rester dans son environnement habituel et donc d'éviter les désorientations et autres troubles du comportement |
| suivi plus régulier des patients |
| Une situation où l’hospitalisation n’améliore pas le pronostic ou le confort du patient |
| traitable en mr |
| Relais médecin généraliste si patient en MRS (traitement adapté et surveillance sur place) |
| Un appel 112 plusieurs heures après que le patient et ou sa famille ait signaler le problème au personnel de la MR |
| Présence d'un projet thérapeutique qui indique clairement que le patient ou sa famille ne souhaite pas d'hospitalisation (soins palliatifs). |

**Supplementary Table S12b:** Open questions (the answers have been translated to English from their original form)

| **How would you define an avoidable hospitalization?** |
| --- |
| Timely intervention by general practitioner, actual desire of patient or family was to stay at home |
| Early care planning not being respected |
| By contacting the general practitioner more quickly, therapies can be implemented more quickly. Through early care planning, a decision can be made not to proceed with hospitalization. |
| Early care planning and DNR registration. Correct involvement of general practitioner. |
| Somewhat more intensive medical care on site is usually not possible due to lack of staff |
| Chronic condition with DNR code is still brought to hospital, admission shortly before weekend or holiday as own general practitioner is not on call |
| An admission resulting from incorrect medical action hours/days before the call and/or failure to respect an existing will/care directive. |
| Quicker contact with the general practitioner and/or specialist so that something can still be resolved or provided during daytime hours. |
| Unnecessary calls, patients who no longer wish to be hospitalized, DNR 3 codes |
| End of life pathology, which can be managed by the general practitioner on site |
| Negative will statement or palliative setting |
| Social admission |
| With a good will statement and a clear plan of action for patients, hospitalization can often be avoided. |
| Comfort care in nursing homes |
| This is an admission that could preferably be done through a day admission/consultation regarding general condition. |
| At the expense of patient comfort, no DNR code present |
| If follow-up by staff and visits by the general practitioner suffice. Additionally, absence of a (realistic) will statement. |
| An admission where the degree and/or intensity of care is the same as could reasonably be provided in the nursing home (if adequate staffing were available). |
| Palliative setting, and family understanding of hopelessness |
| Comfort therapy, proper follow-up by general practitioner/nursing home physician |
| Better care plans, general practitioner who does not come to check |
| No added value of hospitalization on outcome or patient comfort |
| Patients with DNR code and general practitioner who comes on site |
| No added value for patient's quality of life or avoidable through prior extensive discussion between general practitioner and patient/family about therapy limitations |
| Allow the general practitioner to come on site for examination |
| Palliative care |
| Admission that could have been avoided if timely correct medical help had been sought in the patient's care plan or concrete will statements had been drawn up. |
| General deterioration, insufficient staff on weekends in nursing homes |
| Correct palliative care initiated in nursing home |
| Only minor changes to medication necessary before immediately driving to the hospital. Fever does not mean an immediate trip to the hospital... |
| Adequate on-site medication, sufficient nursing staff to administer the correct care. Adequate knowledge of patients and their expected illness. |
| By better coordinating with the general practitioner on how to respond to deterioration or onset of a particular problem. By not waiting until evening, until office hours are over and disciplines are no longer reachable by phone. Many problems can be solved via the clinic and do not need to go through the 112 system. |
| When a very clear DNR policy is known in advance by all parties involved and is effectively followed |
| Patient who can be followed up ambulatory by general practitioner and nurse |
| Better DNR coding for nursing home residents, faster detection of deterioration, better patient status follow-up through more staff and mainly more nursing staff |
| If there is presence of sufficient and properly trained nurses and thus competent nursing staff, the patient should almost always be able to stay in the nursing home. I think, for example, of giving good pain relief, intravenous therapy, setting up a primary palliative team, fall prevention, early recognition of patient becoming ill and responding in a timely manner, preferably by the continuous presence of a (again competent) general practitioner. In other words, creating a kind of hospital environment in a nursing home and mandatory DNR code as soon as patient can no longer be integrated into society (thus for chronic stay with a view to end of life and therefore not applicable to short stay). Together with good and clear agreements towards patient and family. |
| General deterioration |
| Palliative setting |
| When a patient does not want this |
| Mostly chronic pathology |
| Known palliative condition |
| Situations where the general practitioner can also initiate treatment |
| If there had been timely and adequate intervention and the patient had been treated on time in the nursing home, a hospitalization could have been avoided, for example suspicion of a urinary tract infection, faster sampling by nursing staff in the nursing home and timely contact with the general practitioner instead of waiting unnecessarily long. |
| Palliative settings, chronic diseases, DNR |
| When the situation is palliative and these comfort cares can be performed in the nursing home, better policy is necessary here |
| Have realistic expectations |
| Incompetent assessment of severity. |
| transfer to hospital has no value for patients at end of life in |
| Hospitalization that would not have been necessary after clear communication to family and patient to frame expectations regarding treatment and hospitalization. Often there is a demand for hospitalization with unrealistic expectations towards treatment and prognosis of the patient. |
| Therapy limits and long-term prognosis of certain conditions are rarely if ever discussed in detail with family and patient (e.g., consequences of resuscitation, ventilation, dialysis, ... long-term prognosis of dementia, cancer, survival after hip fractures, ...) with all its consequences. |
| better communication between GP/patient/family/nursing care |
| hopeless situation with expected deterioration and plannable DNR and palliative course |
| intervened too late |
| decent DNR policy with agreements with patient and family |
| Follow-up GP |
| An admission is avoidable if the same care can be provided by nursing staff in the residential care centre, under the supervision of the GP. |
| Chronic problem, which can be taken care of in good consultation with the GP and staff in the nursing home. |
| Death of a resident to which a MUG physician attends is completely pointless. |
| DNR 2 or 3 coding, longstanding problem not noticed in time, with a dying patient with only a few hours to live |
| care provided by nurse/caregiver in residential care center |
| Better care in NH, GP to be contacted first, interventions on site |
| Hospitalization due to inadequate primary care/adequate general medicine |
| Where there is actually already a will from patient or family or there should be one and it is not respected by GP or NH staff |
| Admission where patient's wishes are not listened to, progression of condition where insufficiently anticipated, unclear therapy restriction code |
| Sufficient and adequate care |
| A patient who is DNR can stay in the NH and does not have to go to the hospital |
| Futile care |
| At family request, acute decompensation of chronic condition or pain issue (which can be addressed by GP) |
| Clear agreements around therapy restriction. |
| Admissions due to not following up correctly and quickly and insufficient knowledge of patients |
| Admission with a negative will |
| No acute problem or already clear DNR policy signed. |
| Rapidly reversible cause - DNR 2 |
| Admission for which, if prior care planning is properly agreed upon, equally good or sometimes better care can be provided in the nursing home. |
| An admission that should not take place due to faster detection and intervention |
| Planned care |
| DNR code to be discussed with patient and family and optimal treatment in NH without starting treatment in hospital. |
| By thorough examination |
| If adequate DNR coding |
| Treatment by GP possible/ when patient already has therapy limitation but this is not looked at  Not performed if early care planning would have been discussed |
| Hospitalization that would have been avoidable if the general practitioner was available or if a therapeutic plan (should/would this patient still go to hospital for care) had been made beforehand |
| on the one hand sufficiently rapid intervention by nurses so that no escalation of care is necessary, on the other hand more discussion about quality of life so that less pointless care is provided |
| An admission where no additional therapy is started, or necessary investigations to evaluate |
| potentially treatable diagnoses are performed, except comfort therapy which could also be started in the residential care centre. |
| GP follow-up |
| Poor EWS prior to activation |
| prehospital interventions make no sense, people are sometimes too advanced or too old |
| Better care pre-hospital and assessment with treatment |
| correct care plan prepared in advance, such as correct DNR policy, also discussed with family. |
| Pre-existing will, terminal patients, hopeless situation e.g. recurrent admissions |
| If the necessary care can be administered outside the hospital setting, admission is avoidable. |
| Therapy limitation with deterioration |
| When the will is written, no more hospital admissions; inform the GP in time; wait for therapy for >24 hours. |
| More staff in an NH and more visits by GPs would mean that more care could be provided in the NH itself instead of an unnecessary transfer. |
| Comfort care in the NH |
| Knowledge correct patient will, sufficient training of staff to detect alarm signals in a patient |
| Palliative setting: avoidable through good care planning and communication with pt, family and care staff. |
| Better follow up GPs, better discussion DNR coding, clarity of this coding |
| To be seen first by doctor (who knows the patient) instead of referring by phone |
| Pt feels well and prefers to stay in his familiar surroundings. |
| Not only advice by telephone but also visit the patient to see him. |
| With correct end-of-life policy, correct treatment and follow-up, sufficient care supervision, fall prevention,... A definition is not easy, it is a multiplicity of factors. |
| futile care - far-reaching care demand (e.g. intubation, IZ) in patients with significant cognitive deficit. |
| failure to respect DNR |
| Enough comfort and care for the resident in the nursing home |
| Prior DNR coding and adherence to pre-arranged agreements. |
| respect DNR coding, care by prehospital team cf. Physician Response Unit (PRU) |
| An admission that has no impact on improving quality of life over a period of, say, one year. |
| An avoidable hospitalization is one that is technically, logistically and pharmacologically perfectly possible in the residential care centre if properly framed. |
| Palliative policy |
| sometimes admissions of persons even though it is explicitly stated that they no longer desire admission. Sometimes an on-site visit by the family physician can solve a lot. |
| An admission that could have been avoided if earlier adequate treatment in the residential care centre were provided |
| Patient no longer wishes admission or interventions, so comfort care prevails - preventive or ambulatory care would have sufficed. |
| situation in which the patient's clinical picture would have been anticipated at an early stage and/or provision of early care planning in which unnecessary transfer to hospital could have been avoided  timely consultation of the acting physician when symptoms appear, responsible physician who sees patient and consults with family, dares to take medical decisions |
| If family doctor would have visited the patient, an admission can be avoided. |
| Follow-up by general practitioner possible |
| Prevention of hospitalization. |
| Therapeutic persistence. |
| DNR codes not respected. |
| Admission for a chronic deteriorating patient, where in the NH not, or not clearly enough agreements have been made with patient or especially the family around planning of care. Often patients who have not been in the NH for a long time, or who have recently been to the hospital. Usually at night, when the patient is also not well known. So often not yet well-known patients by nursing/caregivers. |
| Patients with extensive DNR policies who cannot be adequately cared for in the nursing home due to infrastructure and staff shortages and where transfer to the hospital only results in comfort care. |
| Well-documented patient wishes |
| By implementing better prevention and medical expertise, many hospitalizations could be avoided and treated in the residential care centre. |
| Patient who could receive treatment there provided enough competent staff. Frequent example: palliative/terminal situations. |
| A hospitalization of such a mild pathology that it can be followed up by the general practitioner, or such a poor prognosis that death can be expected within a few hours and palliative home management can be initiated. |
| Need for examination, admission in accordance with patient wishes and therapy restrictions. |
| - comfort care  - Consultation that can be done by a general practitioner  - consultation that can be done at the hospital |
| (Re)Placement of bladder catheter, file says no hospitalization, consultation with family |
| Situation in which a hospital admission cannot offer any additional value to the patient compared to care that can also be started from the nursing home. |
| Admission that could have been avoided if the general practitioner had already gone there or if clear arrangements had been made with the patient or family beforehand. Need for sufficient supervision, knowledge and personnel so that comfort therapy in residential care centre can also be started. |
| Patient already has DNR and does not meet admission criteria; patient experienced a minor fall but shows no signs of contusion, fracture or cerebral haemorrhage, while MUG was called asking for monitoring |
| Little prospect of improved quality of life on hospital admission |
| more consultation on DNR codes, more medication in nursing homes, more realistic prognosis -- it does concern avoidable MUG calls more than avoidable emergency room admissions |
| deteriorating patient with DNR code who would prefer to die in NH, family does not want this or staff cannot |
| An avoidable hospitalization is when a patient is brought to the hospital with interventions that could be done on an outpatient basis. |
| People who are dying. |
| Initiation of adequate therapy in hospital, with competent and sufficient nursing staff. |
| A call where you can solve the situation on the spot e.g. solve hypoglycaemia. |
| Better follow-up by GP |
| A chronic condition that has been going on for months but suddenly increases on Friday evening? |
| No added value for hospitalization |
| MUG interventions in NH is by definition not good care I think, GP on site, try to solve problems, in case of life threatening acute event, rather solid DNR provided.... |
| At least already DNR coding - yet: is care on site qualitative, humane? |
| Through timely plannable care and end-of-life decisions |
| A non-acute problem that can be addressed properly with the resources available in the outpatient setting (nurse at home, family physician, NH resources). |
| A patient who would not need to go to hospital if the situation had been correctly assessed in time by nursing staff and GP, or if will or care planning was not discussed. |
| Poorly regulated DNR policies and therapeutic persistence with dire prognosis |
| Patient with a DNR 3 policy. Patient with a lingering medical problem without adequate initiation of therapy through the general practitioner. |
| If improvement could have been obtained with timely and adequate care and recognition  this is a hospital admission that can be avoided if there is a clear medical record, clear DNR code, clear early care planning in place so that this can be followed up by the staff in the NH together with the GP. |
| if there is clearly a long run-in with the patient and just no adequate action was taken when it was something minor; or when a family member decides to go against the extended therapy restriction in acute setting |
| less humane care, no added medical value |
| Admissions due to slow deterioration to untenable situation that could have been avoided with early interventions and good follow-up from family physician. |
| Palliative setting, general deterioration without acute deterioration, suspected hip fracture in demented bedridden frail patient where no surgical intervention will happen anyway |
| Care leading to a return to or maintenance of the elderly person in a nursing home, after prior discussion with the attending physician, the patient and, if necessary, the family, with, if necessary, a modification of the patient's local monitoring and, if necessary, his or her treatment. |
| End of life, life project |
| End-of-life wishes or non-curable chronic pathologies poorly treated/recognized in NH |
| Appropriate NH care |
| On-site care possible |
| a clear end-of-life care plan drawn up and regularly reviewed during the stay |
| it all depends on what avoidable means: non-urgent? unjustified? Most of the time, there is no hyperacute event requiring immediate medical intervention and immediate hospital care to guarantee the patient's survival, autonomy, quality of life and dignity. BUT the Belgian healthcare system is not prepared to consider that death is part of the cycle of life and can normally occur in a care setting. |
| frivolous hospitalization or hospitalization made necessary by lack of local care |
| hospitalization that does not improve the patient's state of health or quality of life |
| non-urgent hospitalization, which could be scheduled, or care/treatment that could be carried out by NH staff if clearly instructed by the GP |
| increased nursing home care, enabling patients to remain in their usual environment and thus avoid disorientation and other behavioral disorders |
| more regular monitoring of patients |
| A situation where hospitalization does not improve the patient's prognosis or comfort |
| treatable in NH |
| Relay to the general practitioner if the patient is in NH (appropriate treatment and on-site monitoring) |
| A 112 call several hours after the patient and/or family have reported the problem to the NH staff |
| Presence of a therapeutic plan clearly indicating that the patient or family does not wish to be hospitalized (palliative care) |

EMS: Emergency Medical Services; GP: General Practitioner; NH: Nursing Home; EWS: Early Warning Score; DNR: Do Not Resuscitate

**Supplementary Table S13a**: Open questions (the answers have not been modified from their original version)

| **Please give examples of interventions that could be decided on the spot and could thus avoid hospitalization.** |
| --- |
| Algemeen veel beter communiceren over vroegtijdige zorgplanning door de 1e lijn en het nut van bepaalde type interventies bij bepaalde type patienten.  Bv demente patienten met respiratoire distress. Zou nooit een mug moeten zijn omdat die altijd een DNR 2 code zouden moeten hebben (zinvol medisch handelen). |
| Palliatieve zorgen kunnen ter plaatse uitgevoerd worden dmv opstart van morfine en eventueel midazolam. |
| DNR - Morfine - Palliatie |
| zuurstoftherapie, toediening aerosol, infuus, IV medicatie |
| herplaatsen supra pubische blaassonde, blaassonde, |
| starten van een palliatief / comfort therapie |
| AB opstarten, vocht iv of subcutaan tijdens hittegolf bij gedehydrateerde mensen. |
| Volwaardige beleid van huisartsen, medische materiaal in goede toestand dat de metingen correct kunnen doen |
| antibiotica, palliatieve sedatie |
| Morfine voor de ademhaling, zuurstof, antibiotica |
| Antibiotica - verpleegkundige handelingen zoals transurethale sondespoeling |
| Aërosoltherapie, bloedafname, |
| Eventuele infuustherapie mogelijk in woonzorgcentrum |
| Antibiotica, intraveneus vocht, zuurstoftherapie, pijnstilling. |
| zuurstoftoediening via neusbril, diuretica, antipyretica, antibiotica, ... |
| aanpassen diuretica aan temperatuur omgeving en intake. Opvolgen van early warning systemen voor geriatrie. Op tijd starten van antibiotica. |
| comfortzorg, duidelijke levenseinde besprekingen met WZC |
| Zuurstof, morfine, vocht |
| Zuurstof in rusthuis, antibiotica zo nodig ook IV  Pallatieve sedatie door huisarts |
| zuurstof, comforttherapie en pijnbestrijding |
| Hypoglycemie |
| COPD opstoot, pijnstilling voor rugpijn, delier, ... |
| zuurstoftoediening bij dyspnee |
| Palliatief beleid |
| Antibiotica bij uwi, opdrijven zuurstof bij copd pt’en |
| Anti-emetica bij braken. Eerste hulp bij syncope kennen en kunnen. Zuurstof geven. |
| - Toename oedemen: ophogen diuretica  - Verkoudheid met koorts zonder desaturatie: opstart antibiotica  - UWI: opstart antibiotica  - Pijnklachten na een val waarbij pt nog mobiel is: aanpassing pijnbeleid via staand order  - Niet comfortabel bij DNR 2-3 beleid: via palliatief team staand order om medicatie aan te passen  - Stervende en nood aan sedatie: via huisarts van wacht |
| Extra stimulatie tot drinken, vroegtijdiger adequater antibioticagebruik, duidelijke DNR-codering en open communicatie hieromtrent |
| Opstarten AB, conservatieve behandeling bij uitbehandelde patiënt, comfortzorg ipv therapeutische hardnekkigheid |
| Absoluut! 90% (of zelfs meer) kan vermeden worden. Geven van zuurstof, opstart palliatieve comfortzorg, opstart antibiotica, geven van pijnstilling, geven van vocht… |
| algemene achteruitgang. |
| pijnstilling |
| aerosol, IV vocht |
| behandeling van pneumonie of luchtweginfecties zonder respiratoire distress |
| vochttoediening subcutaan |
| dehydratatie (bij warm weer of dementie mensen meer aanzetten tot voldoende vochtintake), COPD (bij achteruitgang sneller contact opnemen met huisarts voor aanpassing puffers of O2 toediening om een opstoot te vermijden), hartfalen (sneller en meer contact bij huisarts bij gewichtstoename voor aanpassen van vochtafdrijvers), DNR codering (sommige patiënten verkeren in een zeer slechte medische conditie, maar er is dan soms geen DNR codering afgesproken. Indien een patiënt dan een hartstilstand doet wordt dit een muginterventie) |
| zuurstoftoediening |
| Palliatieve ondersteuning, vroegtijdige zorgplanning. O2 toediening, antibiotica. |
| zuurstoftoediening, afwachten werking medicatie |
| Palliatief beleid door HA, adequaat opstarten van medicatie bij COPD opstoot, ... |
| Huisarts contacteren zou veel doen, secundair vervoer instellen voor WZC interventies |
| aerosoltherapie |
| antibiotica, zuurstoftherapie via neusbril, aanmoedigen van voldoende vochtintake |
| wisselen van een supra-pubische sonde of een gewone verblijfsonde |
| als de huisarts al eens kwam kijken en een duidelijk vooraf bepaald DNR-/opname-beleid zou hebben zou dat al veel zijn |
| behandeling koorts, onderzoeken ambulant regelen |
| zuurstoftherapie in het rusthuis |
| Voorbeeld opstart lage hoeveelheid zuurstoftherapie bij Covid-19 bevestigde infectie.  Voorbeeld IV vocht in woonzorgcentrum door verpleging daar te geven bij deshydratatie door chronische diarree. |
| Een huisarts die effectief langskomt in plaats van gewoon het advies te geven tot 112 te bellen. Huisartsen die overleggen met de opnemende disciplines ipv ongeacht het uur door te sturen naar het ziekenhuis. Gebruik van ziektevervoer in plaats van 112. |
| Start antibiotica en pijnstilling  Start comfort zorg |
| - toer van een wondverpleegkundige in het wzc  - huisarts vroegtijdig alarmeren bij afwijkende parameters  - overleg huisarts-geriater voor (acutere) problematiek |
| Antibiotica, pijnstilling, betere communicatie |
| antibiotica voor infecties, tijdelijk zuurstof in wzc, analgesie bij vb heupfractuur (geen MUG nodig voor pijnstilling), |
| De meeste problemen ondervind ik niet met ziekenhuisopnames voor acute medische problemen maar met stervende patiënten (al dan niet met wilsverklaring) die dan ineens tegen wil en dank toch maar naar het ziekenhuis moeten worden overgebracht vaak zonder dat huisarts komt kijken of dat familie hiermee akkoord gaat. Ik ben dan vaak zeer lang bezig met zowel met de huisarts te overleggen (als die al bereikbaar is) als met de familie en vaak valt dan de beslissing om patiënt in het WZC te laten. |
| opstart AB, meer O2 dan 2L, opstart palliatieve sedatie |
| AB opstarten, gepast pijnbeleid |
| Morfine protocols |
| AB voor pneumonie indien duidelijke kliniek. Opstart diuretica bij cordecompensatie. Indien gekende problematiek met exacerbaties vb pijn of COPD met gekende aanvalsmedicatie die nog niet geprobeerd werd |
| Zuurstof toedienen |
| Palliatieve medicatie ter plaatse |
| Geven van koortswerkende middelen |
| ontwateren bij hypertensief longoedeem |
| Hypoglycemie |
| beginnende pneumonie, daling algemene toestand |
| POC labo, antibiotica, opstart IV med |
| plaatsen van iv lijn en bloedafname wanneer de patiënt naar een ander ziekenhuis wordt afgevoerd. Nooit wanner er afvoer is naar ons eigen ziekenhuis. |
| Nogmaals grondig nazicht |
| palliatieve sedatie |
| Zuurstof, antibiotica voor eender welke infectie, iv pijnstillling, iv vocht |
| O2, palliatieve ondersteuning |
| Indien therapeutisch plan gemaakt is voor geen ziekenhuisopname, zou de arts (al dan niet mug) kunnen beslissen om de beste zorgen (bv zuurstof en antibiotica te starten) met een minder precieze diagnose van bv longontsteking |
| toedienen zuurstof |
| Opstarten comforttherapie in palliatieve setting bij ernstige ziekte. |
| zuurstoftoediening, diuretica, vocht |
| duidelijke afspraken op gebied van DNR op voorhand |
| DNR codering! |
| Zuurstof via neusbril, koortswering en antibiotica, morfine bij dnr codering |
| zuurstoftoediening, antibiotica, hechting |
| Aerosol |
| AB R/, aerosol R/ |
| Snelle zuurstof toedienen, aerosol toedienen, ademhalingskine vroeger opstarten, antibiotica opstarten, IV medicatie toedienen indien voorgeschreven door huisarts |
| DNR codes, ab po,… |
| Neen |
| koortspiek, discomfort ikv palliatieve setting, geen huisarts bereikbaar dus geen ondersteuning in zorgvraag zorgpersoneel |
| DNR codes / wisselen sondes /wisselen Katheters |
| Dafalgan bij koorts, algemene achteruitgang, |
| zuurstoftherapie/ koortswering/ pijnstilling |
| IV vocht toedienen voor dehydratatie |
| respect hebben voor afgesproken DNR code, IV/SC vocht toediening in RVT bij dehydratatie, opstart duovent,... |
| sneller en adequater opvolging parameters, correct DNR beleid, zuurstof toediening waar mogelijk, sneller inschakelen HA/CRA arts en meer frequente opvolging |
| - optimaliseren palliatieve zorg  - klinische evaluatie door arts of ervaren vpk vooraleer alarmeren hulpdiensten  - overleg met patiënten en/of familie rond zinvolheid ziekenhuisopname |
| Deshydratatie vermijden |
| Hypo Glyc |
| antibiotica, diuretica, O2 supplement |
| Palliatieve zorg. |
| Opstart pijnstilling, adequate wondzorg |
| AB opstarten, labo's afnemen, herplaatsen SP-sonde, plaatsen van verblijfssonde bij urineretentie |
| opstarten IV medicatie, infuustherapie bij dehydratatie. |
| toediening zuurstof in WZC |
| opvolging vochtbalans om chronische/subacute cordecompensatie te vermijden  mogelijkheid tot IV antibiotica in RVT  voorafgaande therapiebeperking duidelijk besproken met patiënt/familie/huisarts/RVT |
| opstarten palliatieve sedatie bij patienten met DNR codes (indien aanwezig) |
| opstart medicatie door huisarts. Wondzorg door huisarts |
| Palliatieve sedatie |
| Comfortzorgen? |
| Behandeling met AB bij patiënt die ziekenhuisopname wenst te vermijden. |
| Opstart comforttherapie |
| Ondersteundende zuurstoftherapie, antibiotica, plaatsing iv infuus voor vocht bij bv dehydratatie,… |
| Pijnmedicatie toedienen, diuretica, aerosols, … |
| -> Palliatief beleid  -> IV vocht bij bewoners gedurende korte tijd... i.p.v. opname voor iv vocht  -> HOLA |
| AB eventueel intraveneus opstarten. Interventies meer kaderen in wensen van patiënt en therapieberpeking, noodzaak tot opstarten Interventies zorgvuldig afwegen conform wensen en therapiebeperking. |
| - antibioticatoediening bij UWI / bronchitis  - ophogen diuretica ikv corfalen  - plaatsen blaassonde ikv urineretentie |
| Sonde, Aspiratie, O2, ... |
| Comfortzorg, pijnstilling, lage dosis zuurstof |
| Opstart adequate pijnstilling, opstart antibioticatherapie PO, zuurstoftherapie in woonzorgcentrum |
| Antibiotica voor uwi of bronchitis  Toedienen van pijnstilling ikv chronisch probleem |
| Pijnbestrijding/symptoombestrijding, switch van een therapeutische naar palliatief beleid |
| niet altijd ZHopname vermijden, wel mug-interventie vermijden! antibiotica, IV vocht, gewoon deftig klinisch onderzoek |
| sneller opstarten Ab bij pt met blaassonde met vermoeden uwi, zuurstof ophogen bij pt met longproblemen en chronische zuurstof vb ipv 2l naar 4l mogen gaan tijdelijk... |
| Plaatsen nieuwe verblijfsonde door verpleegkundige wzc bij verstopping ipv ambulance bellen.  Preventief werken (bv tijdig verergering hartfalen herkennen en via huisarts nodige medicatie en afspraken regelen ipv ambulance bellen)  Standing orders met huisartsen afspreken bij pijn en misselijkheid ipv ambulance bellen |
| Zuurstoftherapie en antibiotica toedieningen, palliatieve zorgen, vochttoediening via subcutaan infusie. |
| Antibiotica in wzc, iv vocht hij dehydratatie, palliatieve sedatie opstarten makkelijker maken, vervangen van sondes, hechtingen |
| Toediening van zuurstof, medicatie voor hypoglycemie |
| valproblematiek |
| Burinex bij een milde Cor decompensatie. Comfort therapie bij een CBT patient (morfine, enz) |
| Correcte DNR codering, antibiotica toediening, adequate pijnstilling |
| Comfortzorg, antibiotica,... |
| O2 toediening, palliatieve sedatie & comfortzorg, IV medicatie, ..  ik zou eerder de interventies opzoeken waarvoor absoluut een ziekenhuisopname nodig is. Maar alles is afhankelijk van de organisatie van het rusthuis, mogelijkheid tot medische ondersteuning ter plaatse. |
| Duidelijk DNR beleid |
| Aduaat pijnbeleid/comfortbeleid bij patiënten met gekende uitgebreide DNR (zelfs indien zogezegd afgesproken tot geen ziekenhuisopname meer). Vaak geeft dit bij pijn of dyscomfort aanleiding tot een ziekenhuisopname via prehospitaal middelen. |
| Meer zuurstof, palliatief beleid |
| Morfine subbutaan voor comfortzorg in palliatieve situatie, diuretica bij ordecompensatie, antibiotica bij koorts en verwardheid |
| Peri-arrest situatie bij DNR 3 patiënten |
| antibiotica, zuurstof, nitraten, palliatie |
| AB voor pneumonie /cutane infectie - antihypertensiva bij HPT 'opstoot' - diuretica bij hartfalen - |
| palliatieve zorgen, antibiotica voorschrijven |
| zuurstof, comforttherapie, parenteraal toedienen van medicatie |
| Zuurstof geven in het WZC, pijnstilling, bloedname en opvolging door huisarts... |
| Antibiotica voor uwi, antibiotica bij pneumonie, zuurstof therapie, adequate pijnstilling, palliatief beleid |
| antibiotherapie post prelevements, changement de sonde urinaire, soins de plaie, avis d'un specialiste (dermatologue,...), |
| infection,hypoxie,deshydratation,soins palliatifs |
| Antalgiques, traitement hta/oap, soins palliatifs limitation des soins organisé. |
| Administration d’antibiotiques |
| antibiotherapue / exalen non invasifs tel que biologie et examen urines / aerosols/ oxygene |
| soins palliatifs, encombrement à aspirer, broncho-inhalation alimentaire |
| Soins palliatifs |
| oxygénothérapie, soins de confort vs palliatif, administration d'antalgique IM/SC, d'antibiotique, suivi de pathologie chronique via télémédecine, accès plus rapide à un médecin (qui se rend sur place) |
| antibiothérapie et hydratation, soins palliatifs |
| soins de confort, débuter une hydratation SC/IV ou des antibiotiques |
| soins de nursing adéquat et acte confié |
| oxygenotherapie, hydratation iv, antibiotherapie iv, aerosols, sedation |
| atb, aérosol, ... |
| Prélèvement automatique en cas de pyrexie, administration d'oxygène de manière adaptée, traitement des diarhées, ... longue liste |
| Gestion précoce de la bronchopneumonie, infection urinaire qui sont déjà déclarée en sepsis lorsque le 112 se déplace |
| Pose de voies veineuses peripheriques, sondage vesical |

**Supplementary Table S13b**: Open questions (the answers have been translated to English from their original form)

| **Please give examples of interventions that could be decided on the spot and could thus avoid hospitalization.** |
| --- |
| General much better communication about early care planning by first line and the usefulness of certain type of interventions in certain type of patients. Eg demented patients with respiratory distress. Should never be a MUG because those should always have a DNR 2 code (meaningful medical intervention). |
| Palliative care can be performed on site by startup of morphine and possibly midazolam. |
| DNR - Morphine - Palliation |
| Oxygen therapy, aerosol administration, IV drip, IV medication |
| Repositioning supra pubic catheter, bladder catheter |
| Starting palliative/comfort therapy |
| Antibiotics start up, fluids iv or subcutaneous during heat wave in dehydrated people. |
| Full policy of GPs, medical equipment in good condition that can do measurements correctly  antibiotics, palliative sedation |
| Morphine for breathing, oxygen, antibiotics |
| Antibiotics - nursing actions such as transurethral catheter flush |
| Aerosol therapy, blood sampling, |
| Possible infusion therapy in residential care center |
| Antibiotics, intravenous fluids, oxygen therapy, pain relief. |
| Oxygen administration through nasal cannula, diuretics, antipyretics, antibiotics, ... |
| Adjust diuretics to temperature environment and intake. Follow up on early warning systems for geriatrics. Starting antibiotics on time. |
| Comfort care, clear end of life discussions with NH |
| Oxygen, morphine, fluids. |
| Oxygen in nursing home, antibiotics if necessary, also IV |
| Palliative sedation by general practitioner |
| Oxygen, comfort therapy and pain management |
| Hypoglycaemia |
| COPD flare-up, analgesia for back pain, delirium, ... |
| Oxygen administration for dyspnea |
| Palliative management |
| Antibiotics in urinary tract infection, boosting oxygen in COPD patients |
| Antiemetics in vomiting. Know and be able to give first aid for syncope. Giving oxygen. |
| - Increased edema: increase diuretics.  - COPD with fever without desaturation: start antibiotics.  - UTI: start antibiotics  - Pain after a fall where patient is still mobile: adjust pain policy via standing order  - Not comfortable with DNR 2-3 policy: via palliative team standing order to adjust medication  - Dying and need for sedation: via GP on duty |
| Additional stimulation to drink, early adequate antibiotic use, clear DNR coding and open communication about this |
| Initiation of AB, conservative treatment in exhausted patients, comfort care instead of therapeutic persistence |
| Absolutely 90% (or even more) can be avoided. Give oxygen, start palliative comfort care, start antibiotics, give analgesia, give fluids.... |
| Overall deterioration. |
| Analgesia |
| Aerosol, IV fluids |
| Treatment of pneumonia or respiratory infections without respiratory distress |
| Fluid administration subcutaneously |
| Dehydration (in hot weather or dementia encourage people more to take adequate fluid intake), COPD (if deteriorating contact GP more quickly for adjustment of inhalers or O2 administration to avoid a flare-up), heart failure (contact GP more quickly and more frequently if weight gain for adjustment of diuretics), DNR coding (some patients are in very poor medical condition, but DNR coding is then sometimes not agreed upon. If a patient then does cardiac arrest this becomes a MUG intervention) |
| Oxygen administration |
| Palliative support, early care planning. O2 administration, antibiotics. |
| Oxygen administration, awaiting effect of medication. |
| Palliative management by GP, appropriate initiation of medication in COPD flare-up, ... |
| GP contact would do much, set up non-urgent transport for NH interventions |
| Aerosol therapy |
| Antibiotics, oxygen therapy via nasal cannula, encourage adequate fluid intake |
| Changing a supra-pubic catheter or an indwelling catheter |
| If the family physician would already take a look and had a clear predetermined DNR/admission policy that would already be a lot |
| Treat fever, arrange exams on an outpatient basis |
| Oxygen therapy in the NH |
| Example start up low volume oxygen therapy in Covid-19 confirmed infection. |
| Example IV fluid in residential care centre by nursing there to be given in case of dehydration due to chronic diarrhoea. |
| A family physician effectively visiting instead of simply advising to call 112. GPs consulting with admitting disciplines instead of forwarding to the hospital regardless of the hour. Use of non-urgent transport instead of 112. |
| Start antibiotics and analgesia |
| Start of comfort care |
| - tour of a wound nurse in the NH  - Alert GP at an early stage in case of abnormal parameters  - Consult GP or geriatrician for (more acute) problems |
| Antibiotics, pain relief, better communication |
| Antibiotics for infections, temporary oxygen in hospital, analgesia for hip fracture (no MUG needed for pain relief) |
| Most of my problems are not with hospitalizations for acute medical problems, but with dying patients (with or without living will) who suddenly have to be transferred to the hospital against their will, often without the family doctor's permission. It often takes me a long time to consult both the family doctor (if he or she is available) and the family, and often the decision is made to leave the patient in the nursing home. |
| Start AB, more O2 than 2L, start palliative sedation |
| Start AB, appropriate pain management |
| Morphine protocols |
| AB for pneumonia if clear clinic. Start up diuretics if heart decompensation. If known problem with exacerbations e.g. pain or COPD with known attack medication not yet tried |
| Administer oxygen |
| Palliative medication on the spot |
| Giving antipyretics |
| Dehydration in hypertensive pulmonary oedema |
| Hypoglycaemia |
| onset of pneumonia, decline in general condition |
| Point Of Care lab, antibiotics, start IV med |
| Placement of iv line and blood draw when patient is transferred to another hospital. Never when transferred to our own hospital. |
| Again thorough review |
| palliative sedation |
| Oxygen, antibiotics for any infection, iv pain relief, iv fluids |
| O2, palliative support |
| If therapeutic plan made for no hospitalization, physician (MUG or otherwise) could decide to start best care (e.g. oxygen and antibiotics) with less precise diagnosis of e.g. pneumonia |
| Administering oxygen |
| Starting comfort therapy in palliative setting in severe illness. |
| Oxygen administration, diuretics, fluids |
| Clear agreements on DNR in advance |
| DNR coding! |
| Oxygen via nasal cannula, antipyretics and antibiotics, morphine when DNR coding |
| Oxygen administration, antibiotics, suturing |
| Aerosol |
| AB, aerosol |
| Administer oxygen early, administer aerosol, start respiratory physiotherapy earlier, start antibiotics, administer IV medication if prescribed by GP |
| DNR codes, oral AB |
| No |
| Fever peak, discomfort in palliative setting, no GP available so no support for care staff |
| DNR codes / changing urinary catheters / changing catheters |
| Dafalgan (paracetamol) for fever, general deterioration |
| Oxygen therapy/ antipyretic/ analgesia |
| Administer IV fluids for dehydration |
| Respect agreed DNR code, IV/SC fluid administration in nursing home for dehydration, start Duovent,... |
| Faster and more adequate follow-up of parameters, correct DNR policy, oxygen administration where possible, faster GP/nursing home physician referral and more frequent follow-up |
| - optimize palliative care  - clinical evaluation by doctor or experienced nurse before alerting emergency services  - consultation with patients and/or family regarding usefulness of hospitalization |
| Avoiding Dehydration |
| Hypoglycaemia |
| Antibiotics, diuretics, O2 supplement |
| Palliative care. |
| Start up analgesia, adequate wound care |
| Start up AB, labs, reposition suprapubic catheter, place urinary catheter in case of urinary retention |
| Start IV medication, infusion therapy in case of dehydration. |
| Administration of oxygen in hospital |
| Follow-up of fluid balance to avoid chronic/subacute cardiac decompensation |
| Possibility of IV antibiotics in NH |
| Prior therapy limitation clearly discussed with patient/family/home physician/NH |
| Start of palliative sedation in patients with DNR codes (if present) |
| Startup medication by GP. Wound care by general practitioner |
| Palliative sedation |
| Comfort care? |
| Treatment with AB in patient wishing to avoid hospitalization. |
| Start of comfort therapy |
| Supportive oxygen therapy, antibiotics, placement IV drip for fluids e.g. dehydration,... |
| Administer pain medication, diuretics, aerosols, ... |
| -> Palliative policy  -> IV fluids with residents for a short time.... instead of admission for IV fluids  -> Suturing of wounds |
| Start AB intravenously if necessary. Interventions more framed by patient wishes and therapy restriction, need to start interventions carefully considered according to wishes and therapy restriction. |
| - antibiotic administration in case of UTI/bronchitis  - increasing diuretics in case of cardiac failure  - placement of bladder catheter in case of urinary retention |
| Urinary catheter, Aspiration, O2, ... |
| Comfort care, pain relief, low dose oxygen |
| Start up adequate pain relief, start up antibiotic therapy PO, oxygen therapy in residential care center |
| Antibiotics for UTI or bronchitis |
| Administration of analgesia in case of chronic problem |
| Pain relief/symptom control, switch from therapeutic to palliative management |
| Do not always avoid hospitalization, do avoid MUG intervention! antibiotics, IV fluids, just proper clinical examination |
| Faster start up Ab in patient with bladder catheter with suspected UTI, increase oxygen in patient with lung problems and chronic oxygen e.g. instead of just allowing an increase from 2l to 4l |
| Place new indwelling urinary catheter by nursing home staff in case of blockage instead of calling ambulance. |
| Preventive work (e.g. recognizing a worsening of heart failure in time and arranging necessary medication and appointments through the GP instead of calling an ambulance). |
| Standing orders with GPs for pain and nausea instead of calling an ambulance |
| Oxygen therapy and antibiotic administration, palliative care, fluid administration via subcutaneous infusion. |
| Fall problems |
| Burinex (=diuretic) in mild heart failure. Comfort therapy in a DNR patient (morphine, etc). |
| Correct DNR coding, antibiotic administration, adequate analgesia |
| Comfort care, antibiotics,. |
| O2 administration, palliative sedation and comfort care, IV medication, ... |
| I would rather look for the interventions that absolutely require hospitalization. But everything depends on the organization of the nursing home, ability to provide medical support on site. |
| Clear DNR policy |
| Adequate pain policy/comfort policy in patients with known extensive DNR (even if supposedly agreed to no more hospitalization). Often when pain or dyspnea occurs, this results in hospitalization via the EMS. |
| More oxygen, palliative management |
| Morphine subcutaneously for comfort care in palliative situation, diuretics for cardiac decompensation, antibiotics for fever and confusion |
| Peri-arrest situation in DNR 3 patients |
| antibiotics, oxygen, nitrates, palliation |
| AB for pneumonia /cutaneous infection - antihypertensives in hypertensive 'flare-up' - diuretics in heart failure - |
| Palliative care, antibiotic prescribing |
| Oxygen, comfort therapy, parenteral administration of medication |
| Giving oxygen in the NH, pain relief, blood sampling and follow-up by family doctor |
| Antibiotics for UTI, antibiotics for pneumonia, oxygen therapy, adequate pain relief, palliative care policy |
| Antibiotic therapy, urinary catheter changes, wound care, specialist advice (dermatologist,...) |
| Infection, hypoxia, dehydration, palliative care |
| Analgesics, hypertension and heart failure treatment, palliative care, organized care limitation |
| Antibiotic administration |
| Antibiotic therapy / non-invasive tests such as biology and urinalysis / aerosols/ oxygen |
| Palliative care, suction in case of aspiration of food |
| Palliative care |
| Oxygen therapy, comfort care vs. palliative care, administration of IM/SC analgesics, antibiotics, monitoring of chronic pathology via telemedicine, faster access to a physicians (who visit the patient) |
| Antibiotic therapy and hydration, palliative care |
| Comfort care, starting SC/IV hydration or antibiotics |
| Appropriate nursing care and entrusted act |
| Oxygen therapy, iv hydration, iv antibiotic therapy, aerosols, sedation |
| Antibiotics, aerosol, ... |
| Automatic sampling in case of pyrexia, appropriate oxygen administration, treatment of diarrhea, ... long list |
| Early management of bronchopneumonia and urinary tract infections, which are already declared as sepsis when the 112 is en route. |
| Placement of peripheral venous lines, bladder catheterization |

EMS: Emergency Medical Services; NH: Nursing Home; GP: General Practitioner; DNR: Do Not Resuscitate; MUG: Mobile Urgency Group

**Supplementary Table S14**: Emergency Medical System (EMS) in Belgium

| ***Activated level*** | ***Reason for tier activation*** |
| --- | --- |
| MUG 1 | Immediately life- or organ-threatening; highest priority |
| MUG 2 | Potentially life- or organ-threatening, condition requiring urgent medical assistance |
| PIT 3 | Can evolve in the short term to a life- or organ-threatening condition, requiring urgent medical assistance within a known protocol |
| PIT 4 | Can evolve into a life- or organ-threatening condition, which, however, does not require urgent medical assistance |
| Ambulance 5 | Not life- or organ-threatening, but condition requires rapid hospital admission for diagnosis, therapy, or observation |
| GP 6 | Urgent: not life- or organ-threatening, but condition requires rapid medical assessment (within 1-2 hours max) for diagnosis or therapy |
| GP 7 | On-call service: not threatening, but condition requires medical assessment (within the on-call service/12 hours max.) for diagnosis or therapy |
| GP 8 | Scheduled care: referral to the regular/general practitioner outside of on-call service is possible; a new call in case of worsening symptoms is always recommended |

**Supplementary Table S15**: Gantt chart of research plan

***
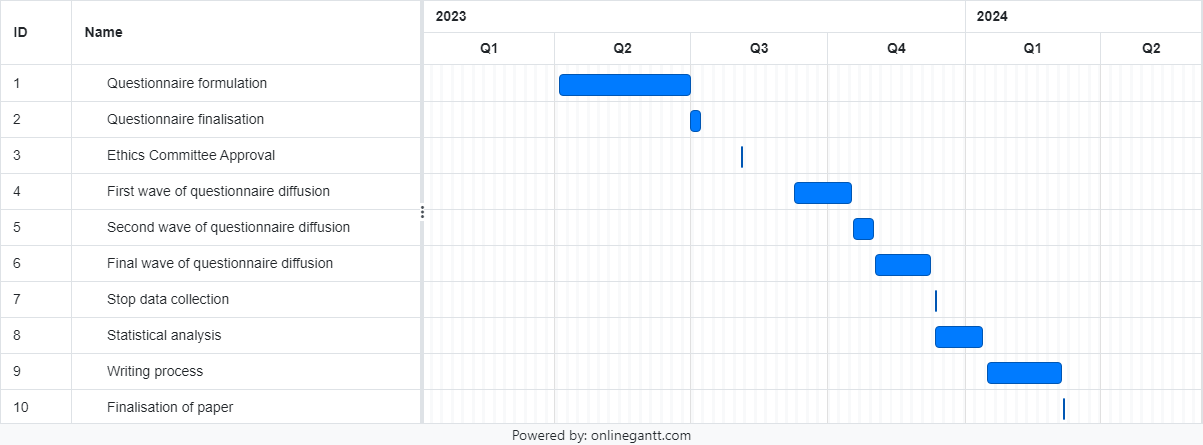
***

1. ***SUPPLEMENTARY FIGURES***

#
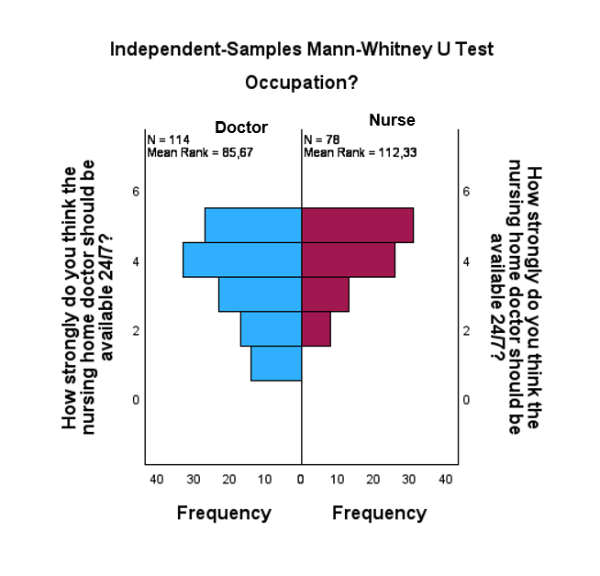


***Fig. 1:*** *How strongly do you think the nursing home doctor should be available 24/7?*


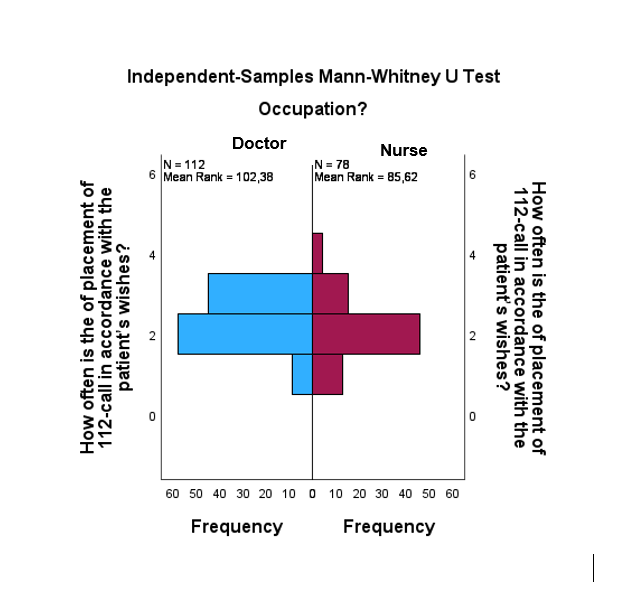


***Fig. 2:*** *How often is the placement of 112-call in accordance with the patient’s wishes?*

#
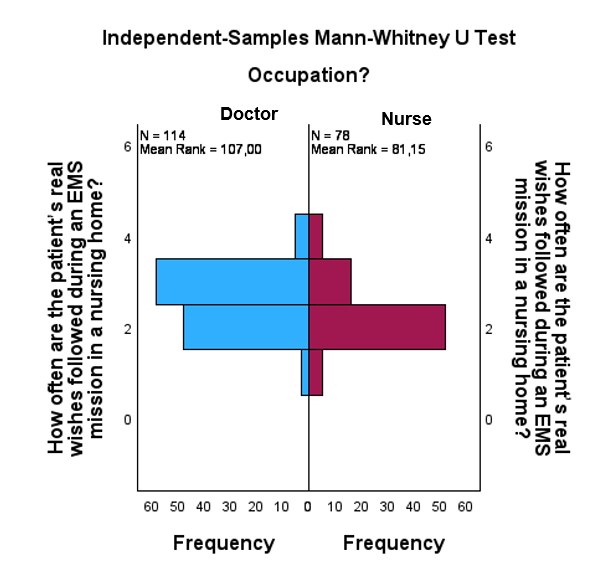


# ***Fig. 3:*** *How often are the patient’s real whishes followed during an EMS mission in a nursing home?*


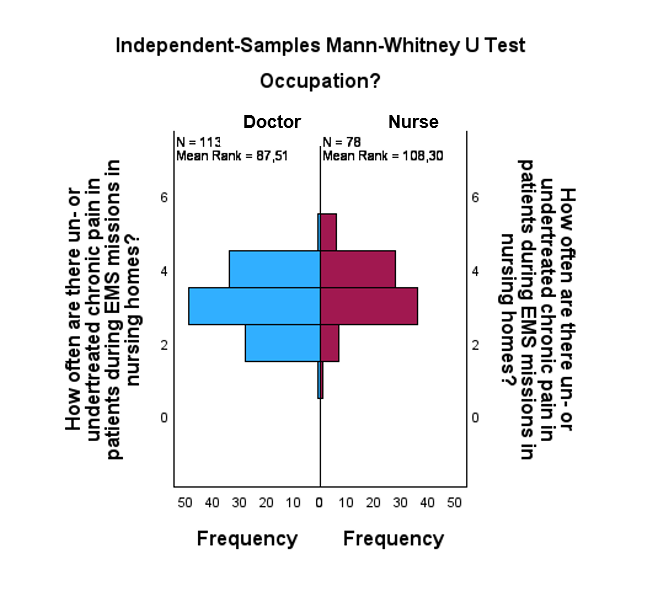


***Fig. 4:*** *How often are there un- or undertreated chronic pain in patients during EMS missiong in nursing homes?*


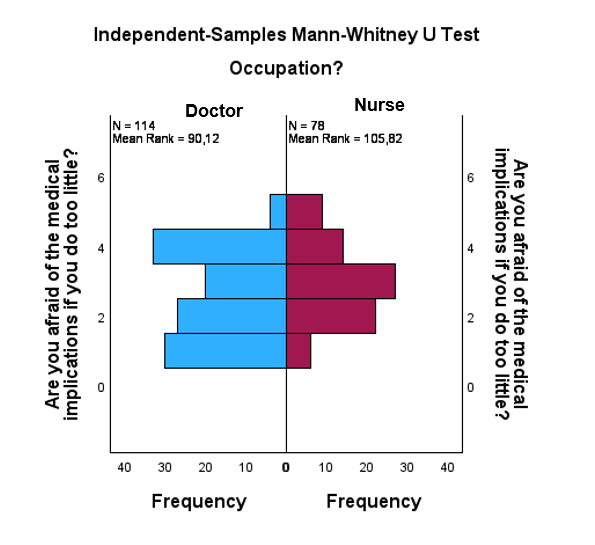


***Fig. 5:*** *Are you afraid of the medical implications if you do too little?*


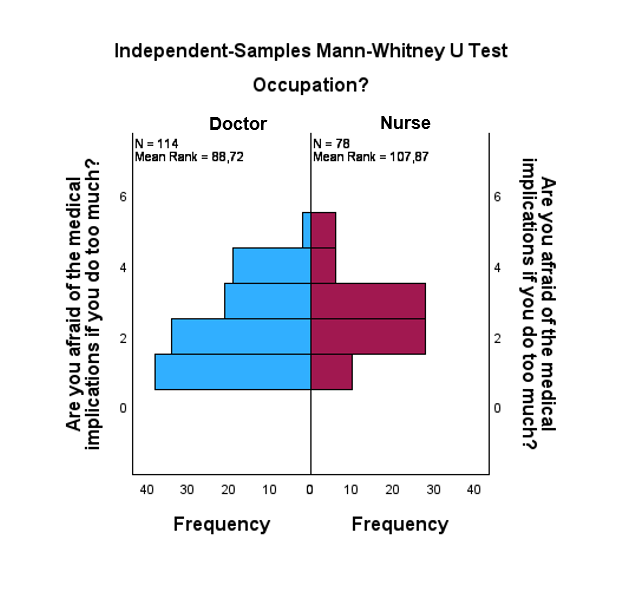


***Fig. 6****: Are you afraid of the medical implications if you do too much?*

#
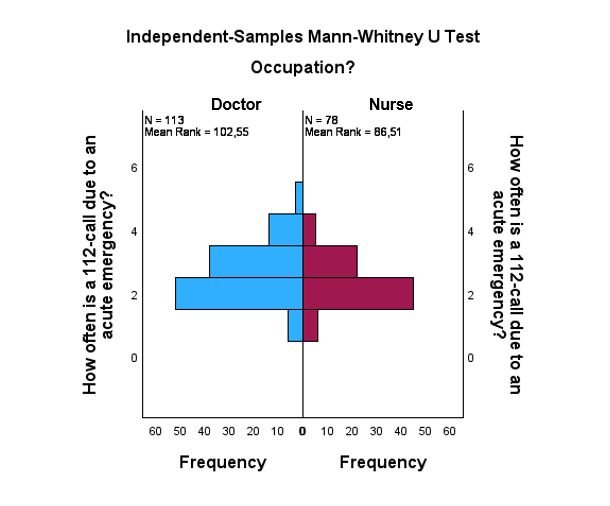


***Fig. 7:*** *How often is a 112-call due to an acute emergency?*


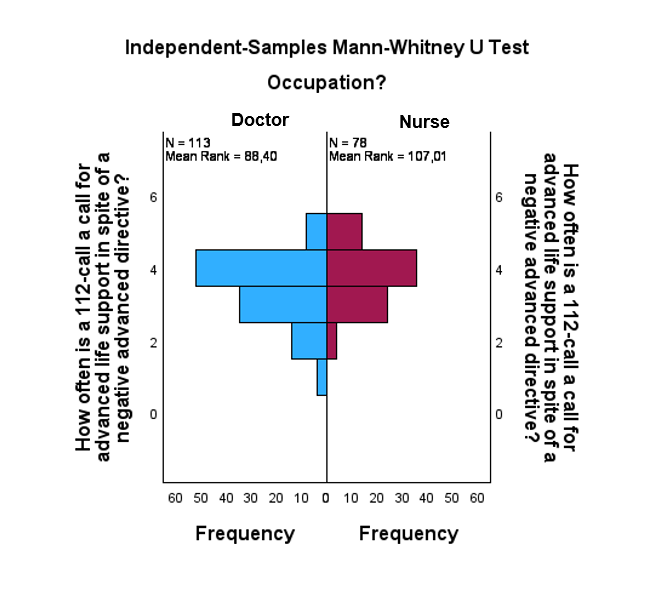


***Fig. 8:*** *How often is a 112-call a call for advanced life support in spite of a negative advance directive?*


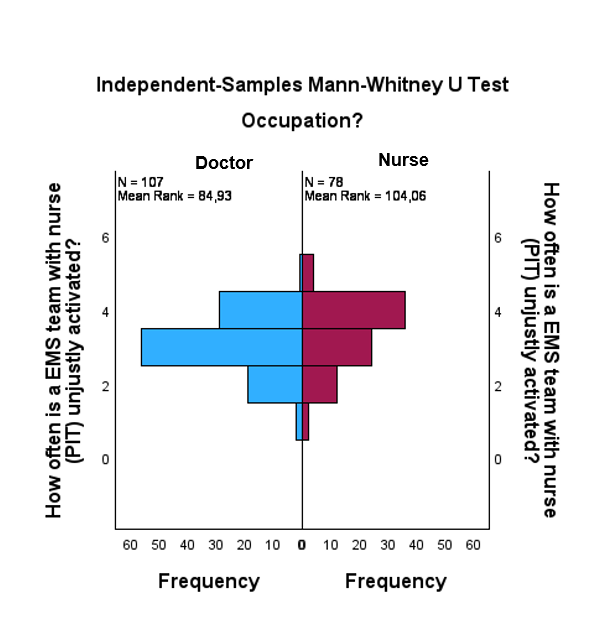


***Fig. 9******:*** *How often is a EMS team with nurse (PIT) unjustly activated?*
