# Supplementary material for: Emergency physicians’ and nurses’ perception on the adequacy of emergency calls for nursing home residents: a non-interventional prospective study
Source: Front Med (Lausanne). 2024 Jun 19;11:1396858. doi: 10.3389/fmed.2024.1396858 (PMC11220277; doi:10.3389/fmed.2024.1396858)

***SUPPLEMENTARY MATERIAL***

**SUPPLEMENTARY GRAPHS**

**Supplementary Graphs G1:** Characteristics of the respondents

1. **Graph G1a:** Area of work?


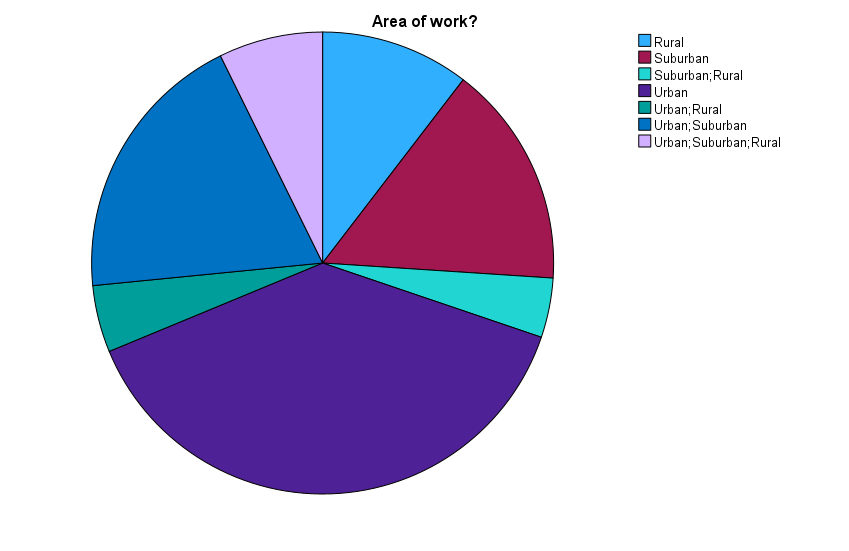


1. **Graph G1b:** Characteristics of the respondents – Occupation?


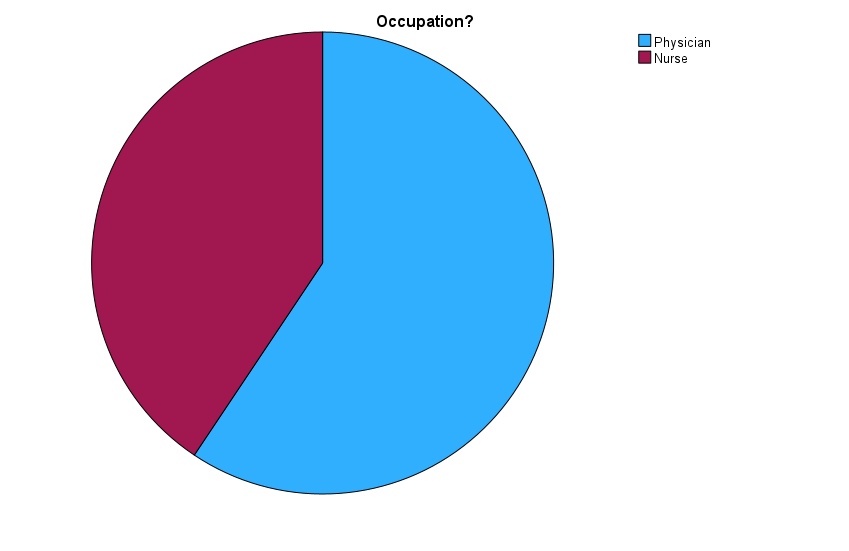


**Supplementary Graphs G2:** Characteristics of Emergency Medical Services (EMS) interventions in nursing homes (general information)

1. **Graph G2a:** Are there more EMS interventions during weekends and nights?


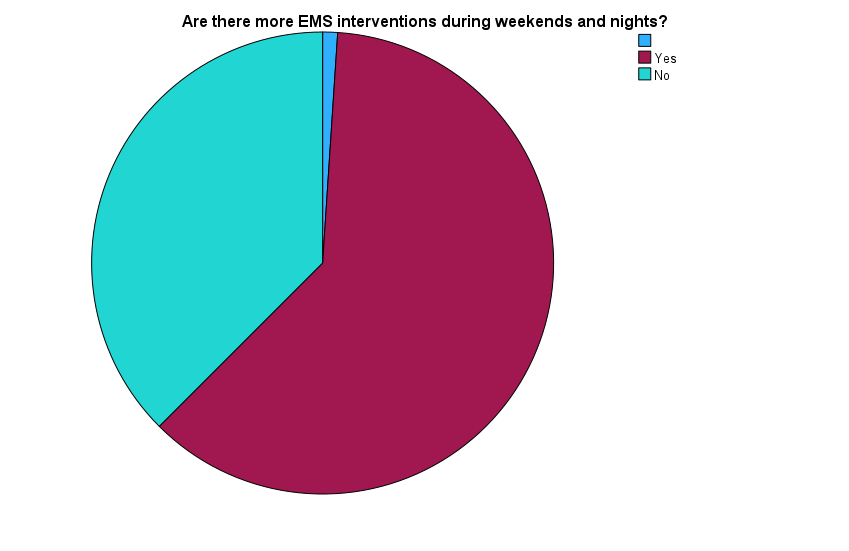


1. **Graph G2b:** Is there sufficient personnel in nursing homes?


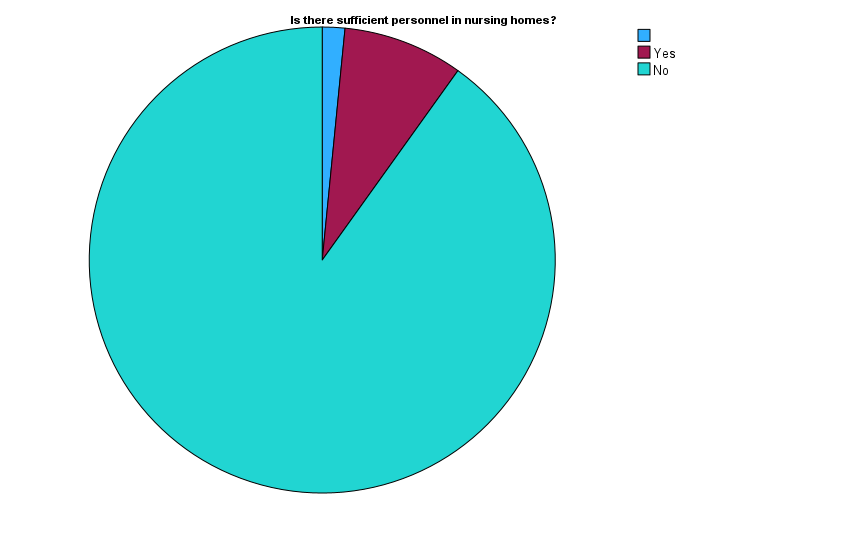


1. **Graph G2c:** Is there sufficient competent personnel in nursing homes?


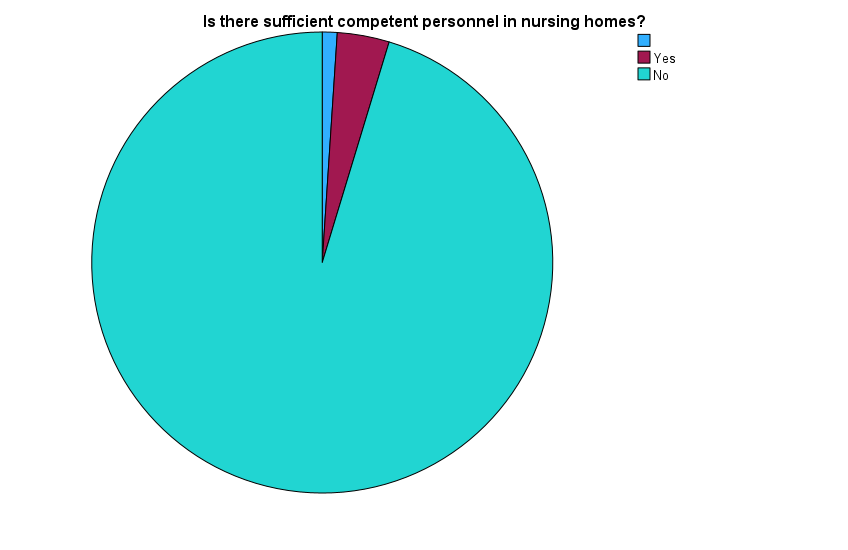


1. **Graph G2d:** Is there a difference in staffing during nights and weekends?


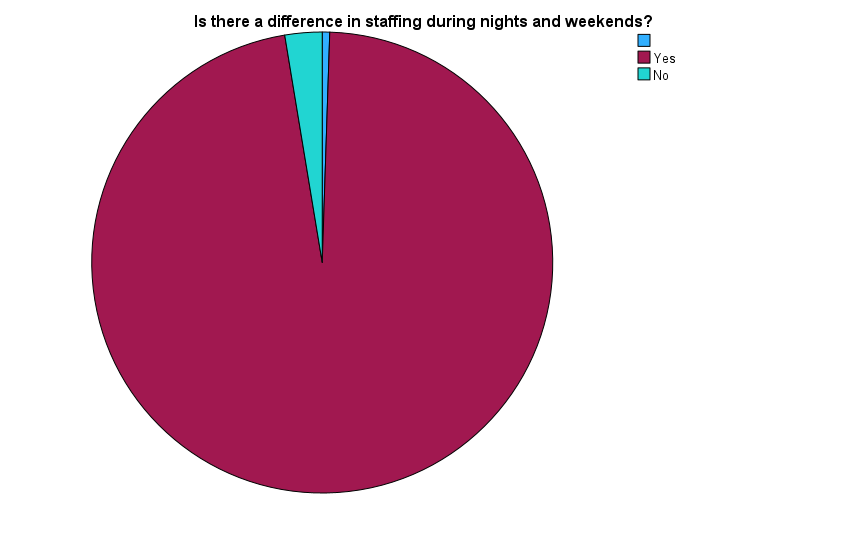


**Supplementary Graphs G3:** Characteristics of EMS interventions in nursing homes (specific information)

1. **Graph G3a**: Characteristics of EMS interventions in nursing homes (specific information) – 112-caller?


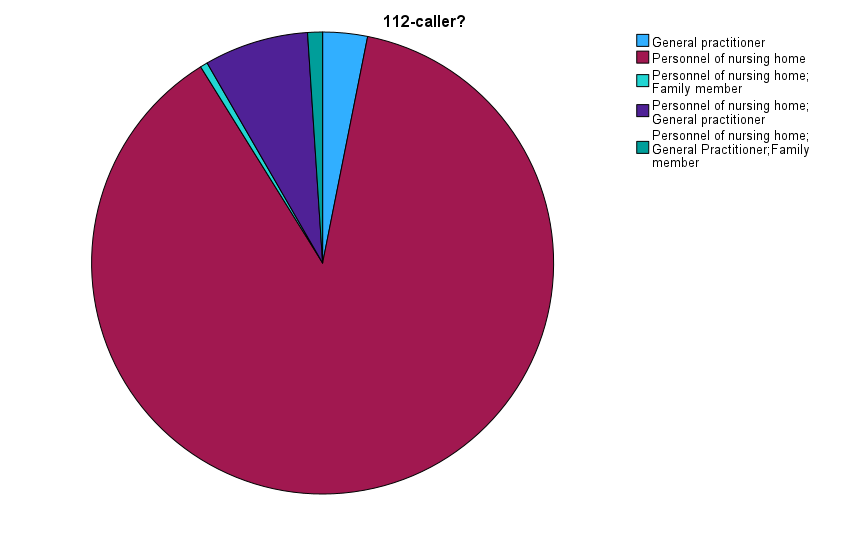


1. **Graph G3b**: How often is there telephone contact with a general practitioner before the initiation of a 112-call?


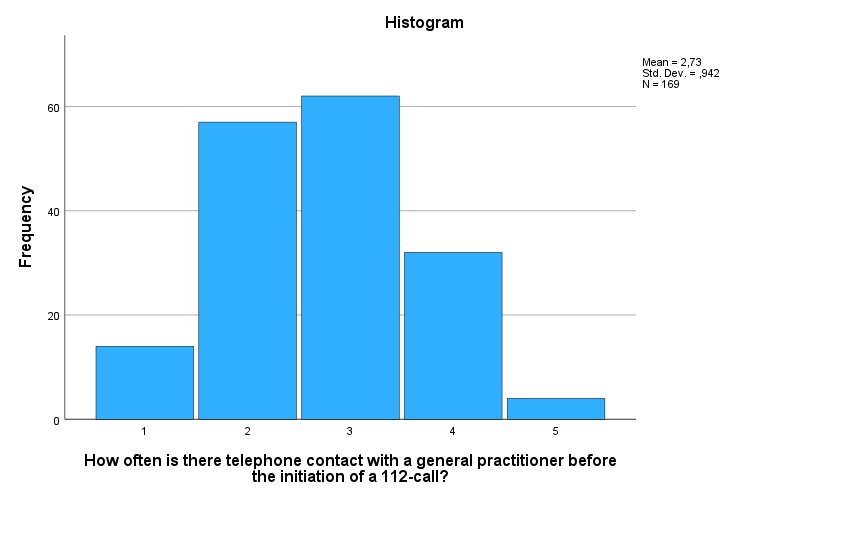


1. **Graph G3c**: How often is the general practitioner present before the initiation of a 112-call?


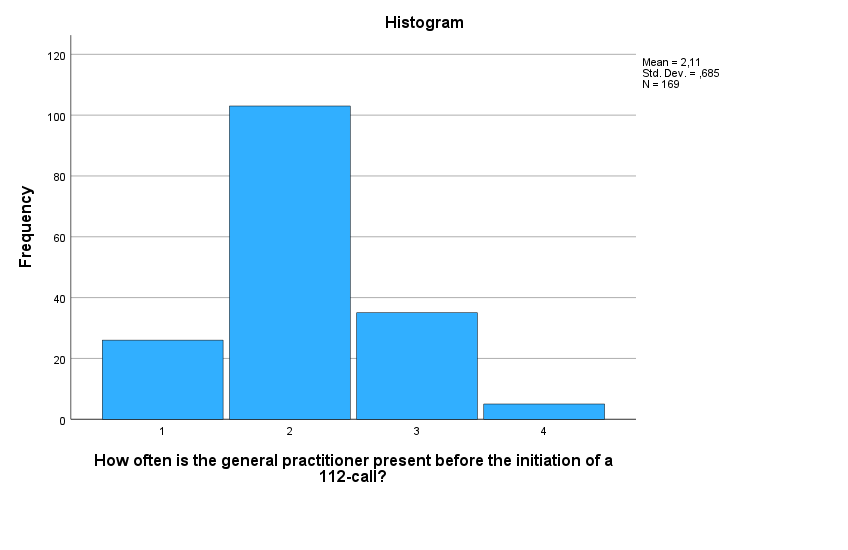


1. **Graph G3d**: How often is the placement of a 112-call in accordance with the patient’s wishes?


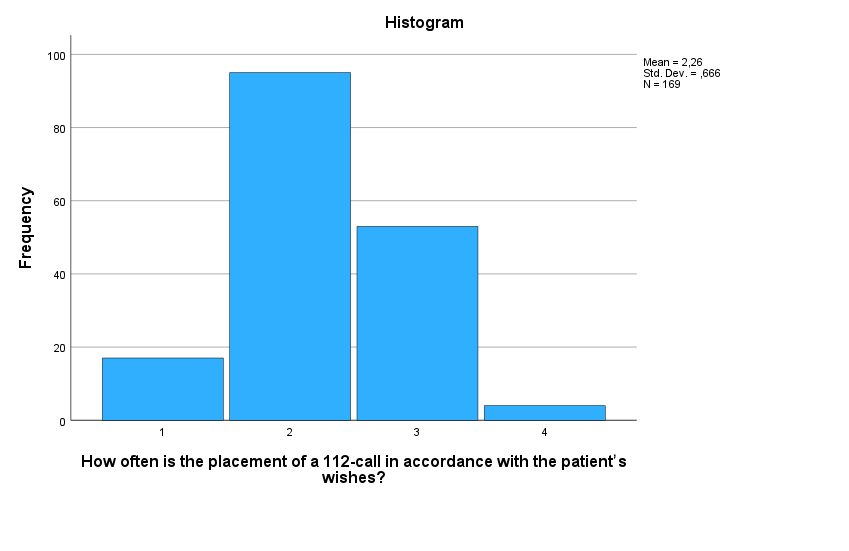


1. **Graph G3e**: How often is a 112-call due to an acute emergency?


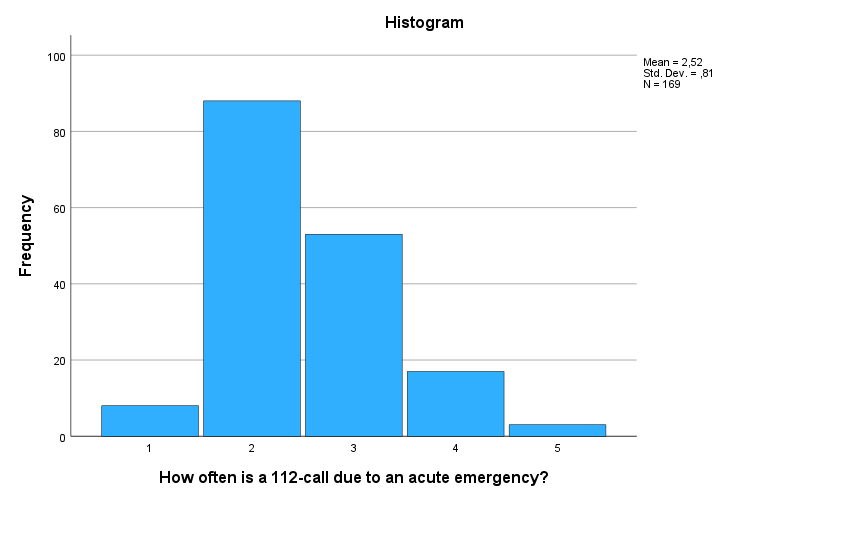


1. **Graph G3f**: How often is a 112-call due to a slowly deteriorating chronic condition?


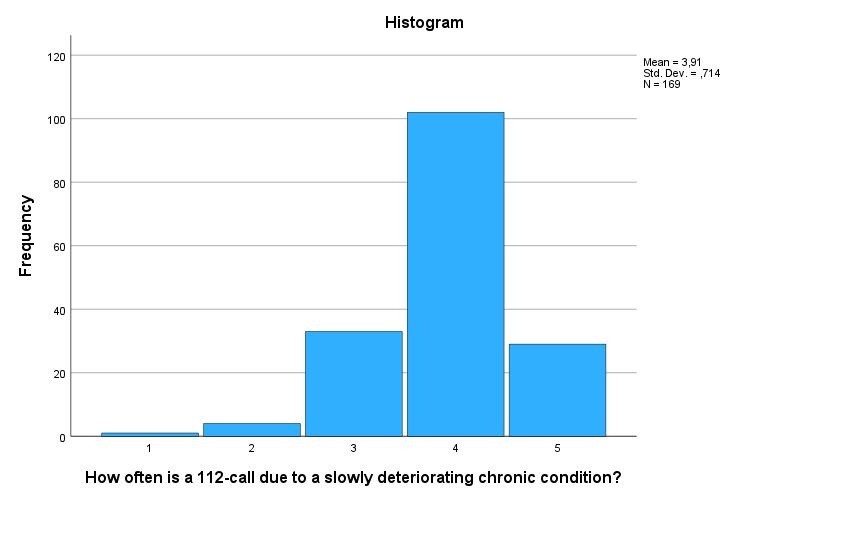


1. **Graph G3g**: How often are EMS interventions in nursing homes really necessary or indicated?


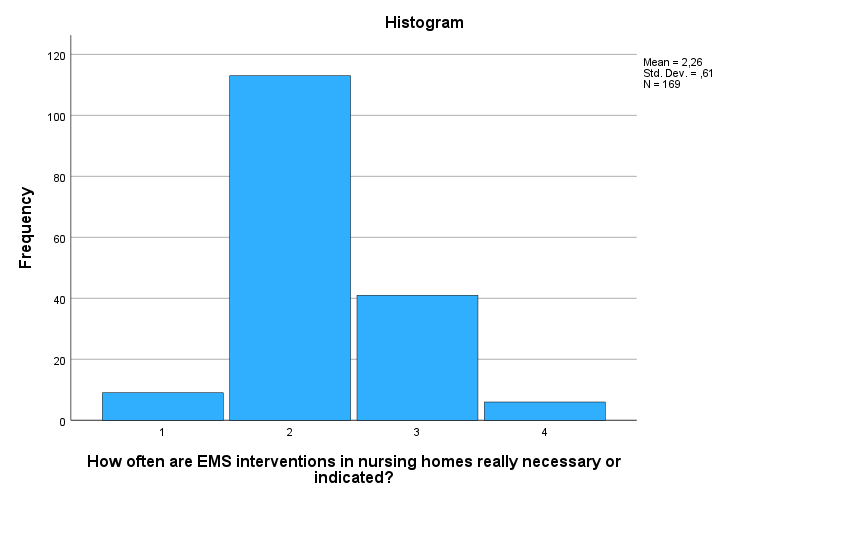


1. **Graph G3h**: How often is the appropriate EMS level activated?


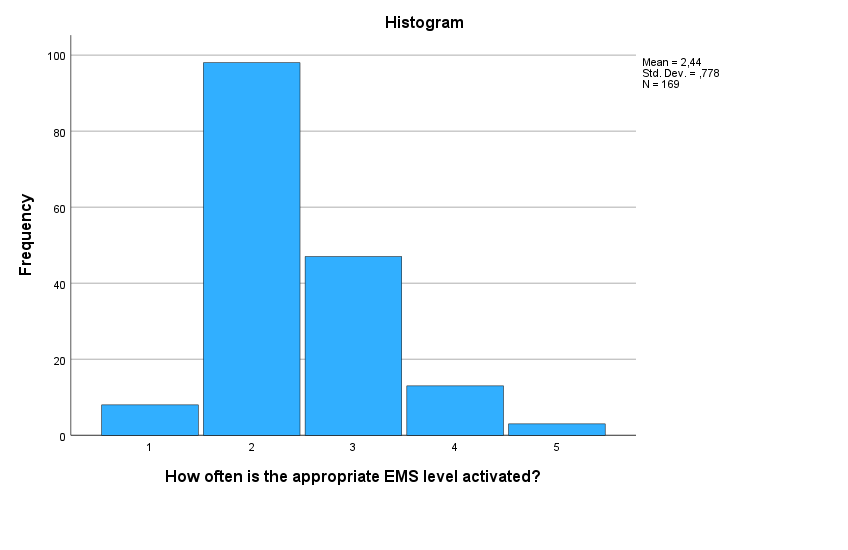


1. **Graph G3i**: How often are hospitalizations avoidable?


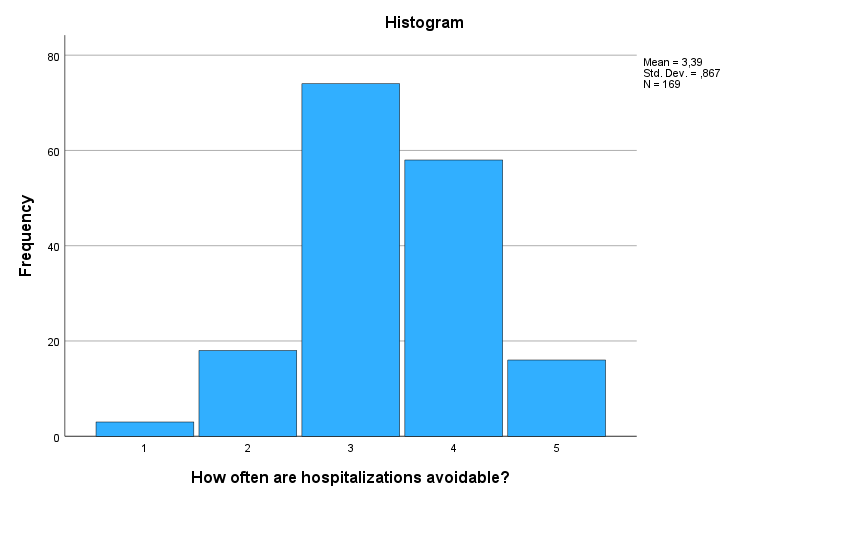


1. **Graph G3j**: How often are the patient’s real wishes followed during an EMS mission in a nursing home?


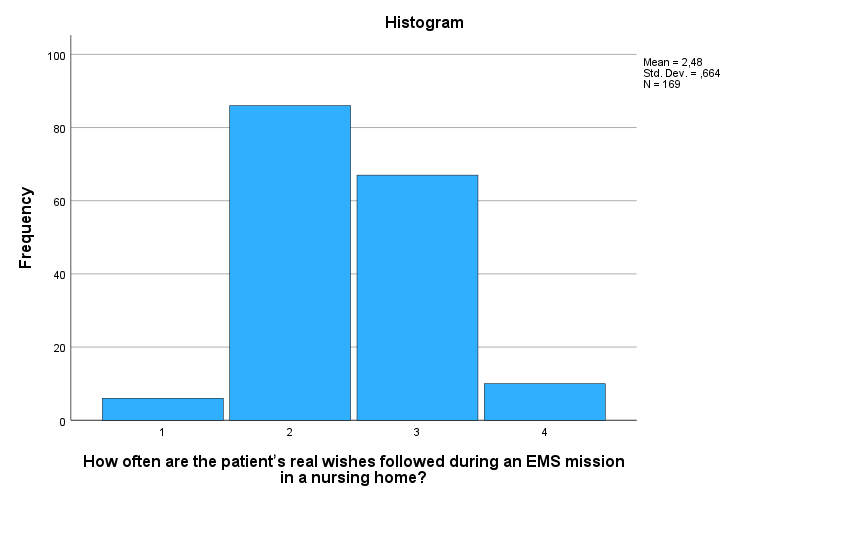


1. ***Graph G3k:*** *How often is a 112-call due to the unavailability of the general practitioner, nursing home physician or on call physician?*

*
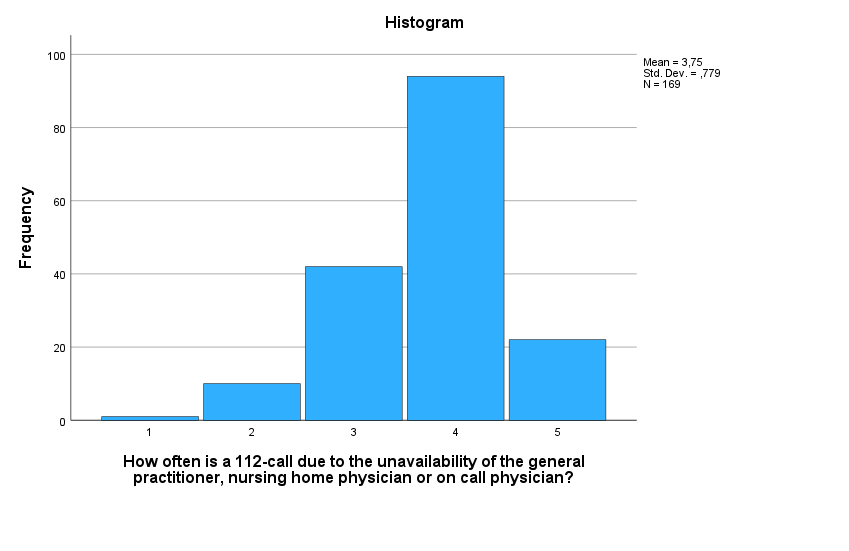
*

**Supplementary Graphs G4:** Medical interventions

1. ***Graph G4a:*** *How often are medical interventions performed by the EMS team (any member) during nursing home interventions?*


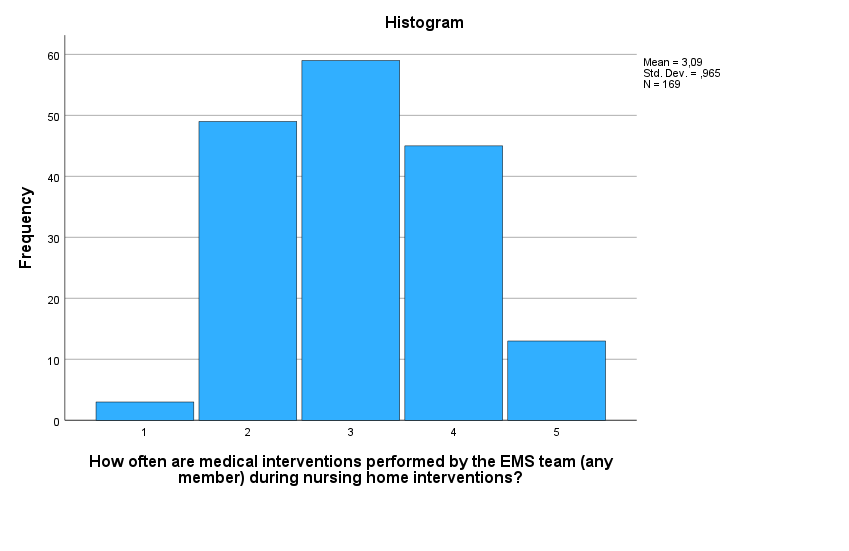


1. ***Graph G4b:*** *How often are medical interventions performed by a physician during nursing home interventions?*

*
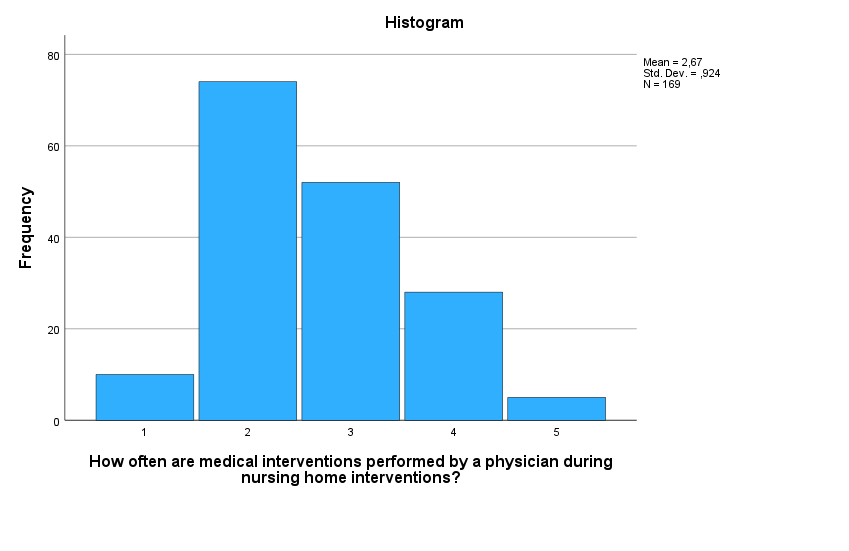
*

1. ***Graph G4b:*** *Do you think hospitalizations could be avoided if the nursing home staff had more authority concerning the initiation of such medical intervention?*


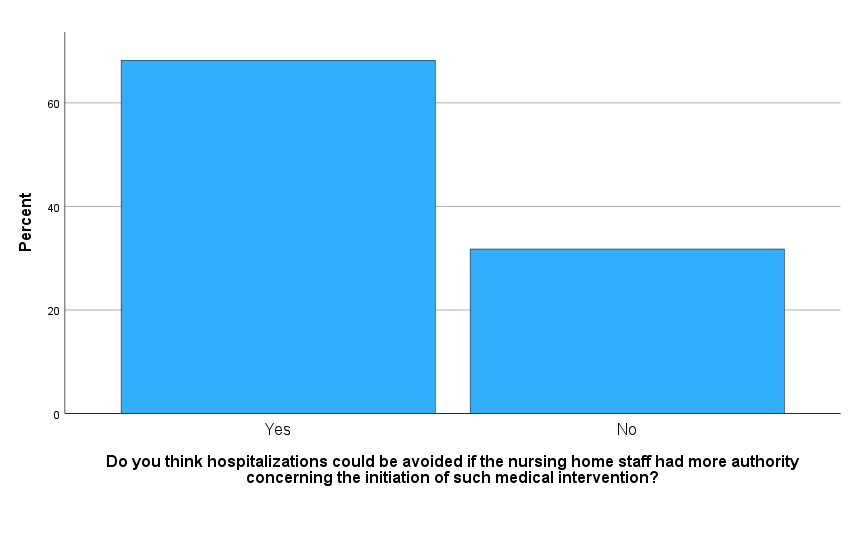


**Supplementary Graphs G5:** Advances directives

1. ***Graph G5a:*** *How often is there an advance directive available in the nursing home?*


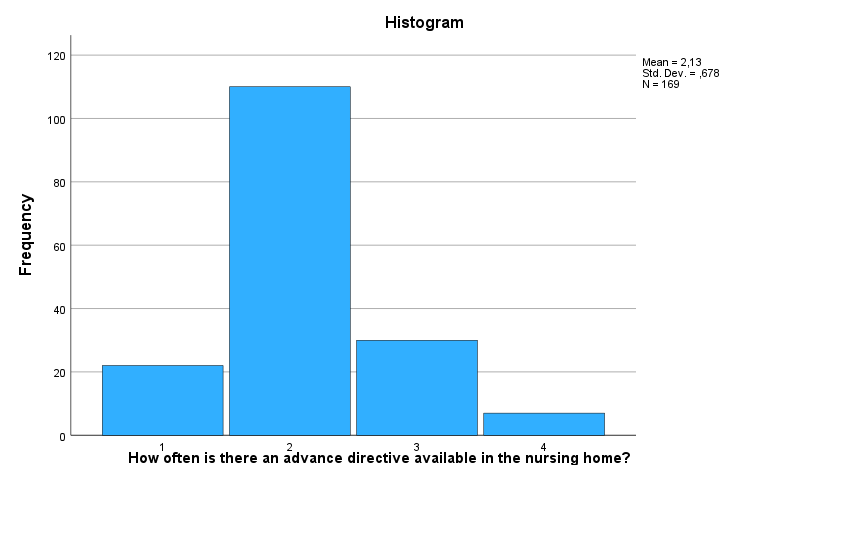


1. ***Graph G5b:*** *How often is a 112-call a call for advanced life support in spite of a negative advance directive?*


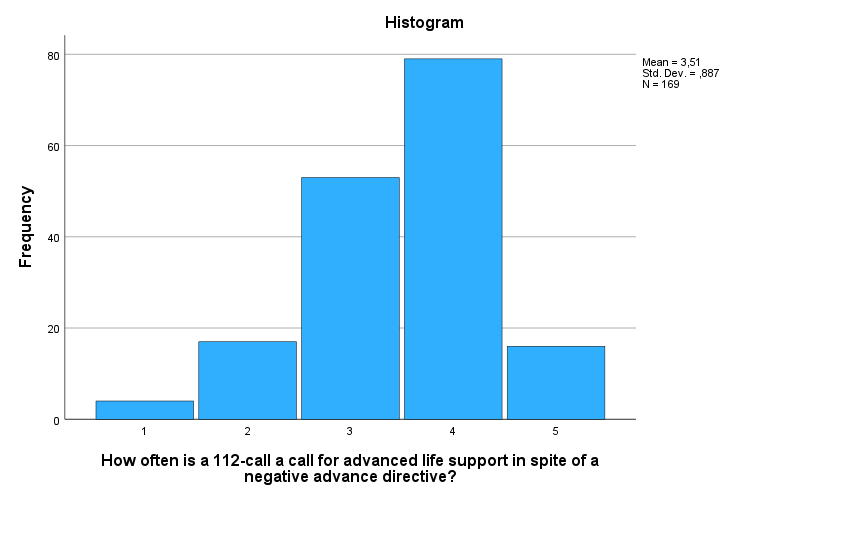


1. ***Graph G5b:*** *How adequate is palliative care in nursing homes?*


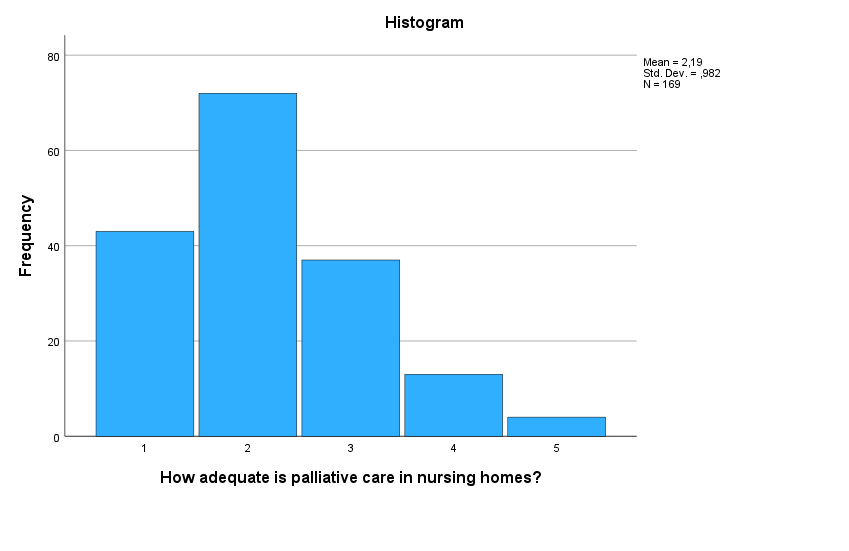


1. ***Graph G5b:*** *How often are there un- or undertreated chronic conditions in nursing home residents during EMS interventions in nursing homes?*


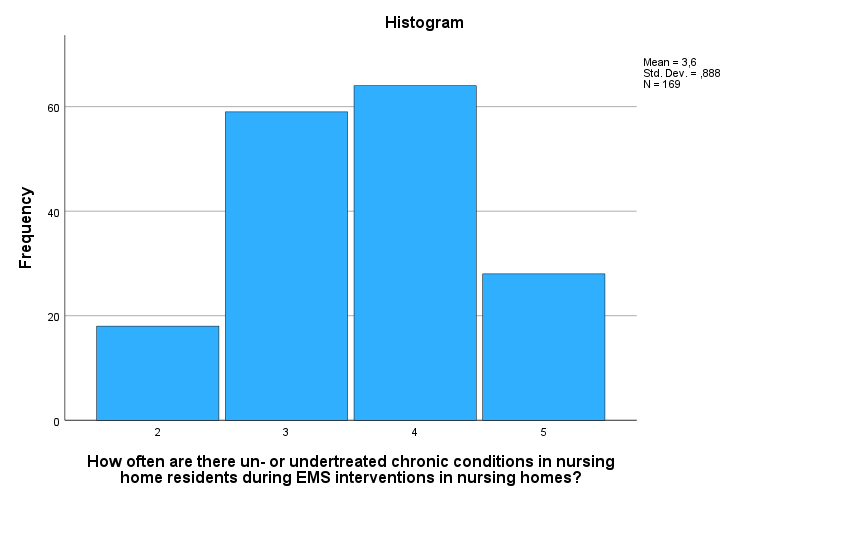


**Supplementary Graphs G6:** Emotions

1. ***Graph G6a:*** *Are you afraid of the medical implications if you do too little?*


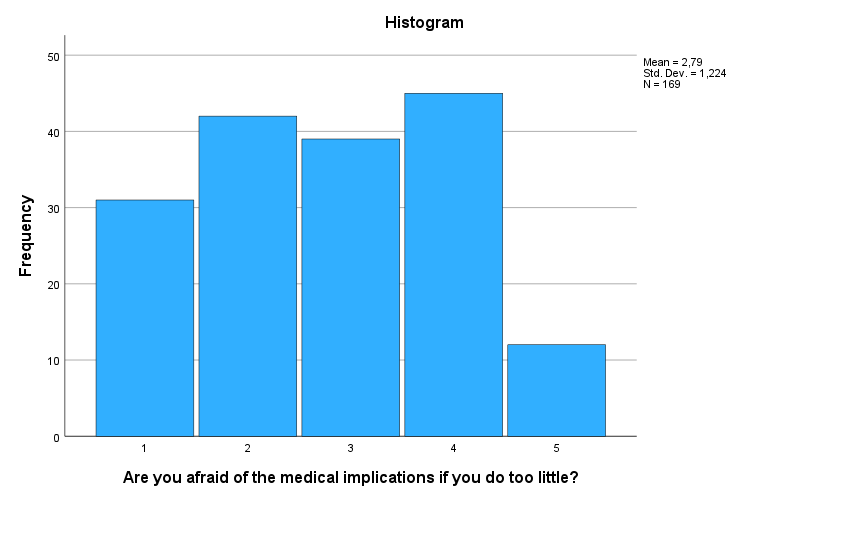


1. ***Graph G6a:*** *Are you afraid of the medical implications if you do too much?*


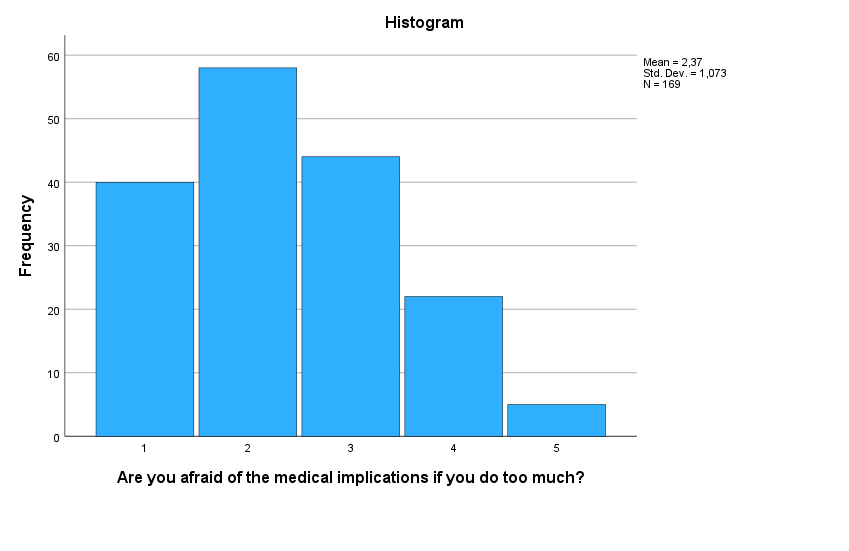


1. ***Graph G6a:*** *How often does the 112-call shift the problem away from the nursing home personnel towards someone else?*


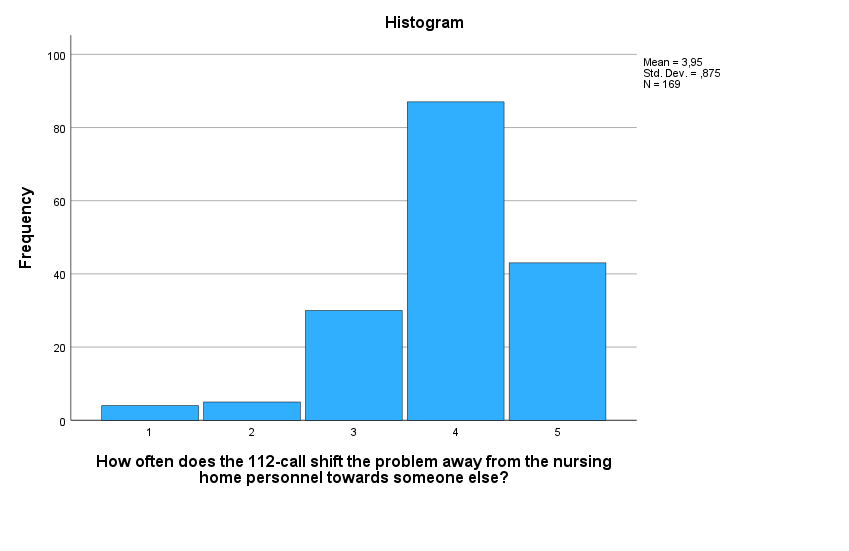


1. ***Graph G6a:*** *How often does hospitalization shift the problem away from the EMS team towards someone else?*


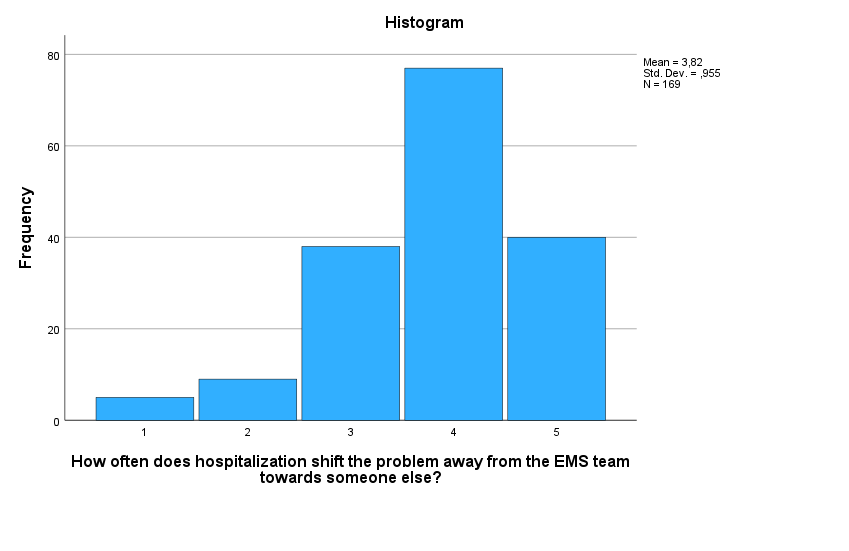

Supplement: Supplementary file 3 [file Data_Sheet_3.docx]
